# Viability of HepG2 and MCF-7 cells is not correlated with mitochondrial bioenergetics

**DOI:** 10.1038/s41598-023-37677-x

**Published:** 2023-07-04

**Authors:** Judit Doczi, Noemi Karnok, David Bui, Victoria Azarov, Gergely Pallag, Sara Nazarian, Bence Czumbel, Thomas N. Seyfried, Christos Chinopoulos

**Affiliations:** 1 Institute of Biochemistry and Molecular Biology, Department of Biochemistry, Semmelweis University, Budapest, 1094 Hungary; 2 Biology Department, Boston College, Chestnut Hill, MA 02467 USA

**Keywords:** Biochemistry, Cancer

## Abstract

Alterations in metabolism are a hallmark of cancer. It is unclear if oxidative phosphorylation (OXPHOS) is necessary for tumour cell survival. In this study, we investigated the effects of severe hypoxia, site-specific inhibition of respiratory chain (RC) components, and uncouplers on necrotic and apoptotic markers in 2D-cultured HepG2 and MCF-7 tumour cells. Comparable respiratory complex activities were observed in both cell lines. However, HepG2 cells exhibited significantly higher oxygen consumption rates (OCR) and respiratory capacity than MCF-7 cells. Significant non-mitochondrial OCR was observed in MCF-7 cells, which was insensitive to acute combined inhibition of complexes I and III. Pre-treatment of either cell line with RC inhibitors for 24–72 h resulted in the complete abolition of respective complex activities and OCRs. This was accompanied by a time-dependent decrease in citrate synthase activity, suggesting mitophagy. High-content automated microscopy recordings revealed that the viability of HepG2 cells was mostly unaffected by any pharmacological treatment or severe hypoxia. In contrast, the viability of MCF-7 cells was strongly affected by inhibition of complex IV (CIV) or complex V (CV), severe hypoxia, and uncoupling. However, it was only moderately affected by inhibition of complexes I, II, and III. Cell death in MCF-7 cells induced by inhibition of complexes II, III, and IV was partially abrogated by aspartate. These findings indicate that OXPHOS activity and viability are not correlated in these cell lines, suggesting that the connection between OXPHOS and cancer cell survival is dependent on the specific cell type and conditions.

## Introduction

Metabolic reprograming for acquiring nutrients to satisfy bioenergetic, biosynthetic, and redox demands is a hallmark of cancer^1,2^. This reprograming is at least partly due to an adverse tumor microenvironment characterized by hypoperfusion and thus, limited [O_2_] partial pressure^3^. Intratumoral hypoxia has fuelled the notion that oxidative phosphorylation (OXPHOS) of cancer cells may be dysfunctional^4^ the severity of which depend on the actual level of oxygen concentration^5^. To this end, however, research directives in the past two decades support opposite claims, namely that OXPHOS is either entirely defective^6^ and this assists growth and metastasis^7^^,^^8^ or that cancer cells exhibit normal or even enhanced OXPHOS capacity^9^^,^^10^. More refined statements addressing the role(s) of individual OXPHOS components -such as that of complex I- in cancer cell survival also suffer from the same antipodicity^11^.

Here we used two of the most widely used cancer cell lines, HepG2 and MCF-7, each cited in > 300,000 entries (https://bit.ly/3S5FWee and https://bit.ly/3LfsvWF). Regarding HepG2, originally thought to be a cell culture model of hepatocellular carcinoma, it is now known that these cells rather mimic hepatoblastomas^12^. Nonetheless, it is a popular hepatic cell line that does not exhibit sublineality^12^. Furthermore, HepG2 cultures do not exhibit great metabolic variability, thus they are deemed as suitable for real-time assessment of mitochondrial toxicity^13^.

On the other hand, MCF-7 cells exhibit strong sublineality maintained by the presence of small amounts of estrogen in the fetal bovine serum, thus being at the mercy of the vendor and batch-to-batch variability^14^. In addition to that, MCF-7 cells sublines exhibit differences in copy number alteration profiles, affecting even the most basic aspects of cell phenotype^15^. Nonetheless, most laboratories -except the group of Lisanti^16–18^, and another group in China^19^- concur on the findings that MCF-7 cells respire minimally^20–26^. Somewhat higher (but still moderate) respiration- and extracellular acidification rates of MCF-7 cells in response to serum treatment, a phytochemical and estrogen receptor α activation have been published by the group of Klinge^27–29^.

We report that HepG2 cells exhibit robust oxygen consumption rates (OCRs) and respiratory capacity, implying OXPHOS-capable in situ mitochondria; yet, inhibition of any of the respiratory complexes leading to a complete loss of respiration, or severe hypoxia or uncoupler-conferred complete collapse in mitochondrial membrane potential (∆Ψm) exerted almost no effect on their viability. On the other hand, MCF-7 cells exhibited minimal respiratory capacity implying OXPHOS-defective in situ mitochondria; however, some RC inhibitors, severe hypoxia or maximal uncoupling led to a considerable loss of MCF-7 cells viability. Furthermore, MCF-7 cells also showed a non-mitochondrial component consuming oxygen. As no pattern emerged, thus no correlation between OXPHOS capacity and cell viability could be established, we posit that the reliance of tumor cells to OXPHOS for survival is cell- and condition-dependent. We further caution that regarding the relation of mitochondrial oxidative phosphorylation and survival no safe extrapolations can be made from one cancer cell type to another.

## Materials and methods

All methods were carried out in accordance with relevant guidelines and regulations of the Semmelweis University. All methods reported are in accordance with the ARRIVE guidelines (https://arriveguidelines.org).

### Cell cultures

HepG2- and MCF-7 cells were grown in Dulbecco’s Modified Eagle Media and in Minimum Essential Medium Eagle, respectively. All cultures were supplemented with 10% fetal bovine serum, 2 mM glutamine and kept at 37 °C in 5% CO_2_. All media were further supplemented with penicillin, streptomycin and amphotericin. Experiments were carried out at day 1–2 post plating, unless stated otherwise.

### Isolation of mitochondria from cell cultures

Cells were harvested by scraping and centrifuged (2 min, 3,500 rpm, 4 °C). The pellet was suspended in a solution containing 225 mM mannitol, 75 mM sucrose, 5 mM HEPES, 1 mM EGTA, 1 g/l bovine serum albumin pH 7.4, centrifuged and the supernatant was discarded. The pellet was resuspended in the same buffer and homogenised with 10 strokes of A and 5 strokes of B pestle in a Dounce homogeniser. The homogenate was centrifuged twice (10 min, 600 g, 4 °C) and the pellet was discarded. Afterwards the supernatant was centrifuged (10 min, 14,000 g, 4 °C). The supernatant was discarded and the pellet was resuspended in the original media that was saved.

### OXPHOS complex assays

10^5^ cells were seeded in 25 cm^2^ flasks (for complexes II, III, IV) and after 1 day (or upon reaching 90% confluence for complex V) the culture medium was exchanged with a solution the composition of which was: 120 mM NaCl, 3.5 mM KCl, 20 mM HEPES, 1.3 mM CaCl_2_, 1 mM MgCl_2_, 10 mM glucose, 4 mM glutamine, pH 7.4 with or without OXPHOS inhibitor (5 µM rotenone, 1 µM atpenin A5, 1 µM myxothiazol, 1 mM NaN_3_, or 5 µM oligomycin). For CI measurement cells were seeded in 175 cm^2^ flasks and grown to near confluence before medium change. After 24 h, 48 h or 72 h of incubation, cells were harvested by scraping, centrifuged (2 min, 3500 rpm, 4 °C) and resuspended in a lower volume of their respective supernatant except for CI measurement, where isolated mitochondria were prepared. Samples were permeabilized by 3 freeze–thaw cycles. Complex I (CI), Complex II (CII), Complex III (CIII) and Complex IV (CIV) assays were performed as described in^30^ and^31^ with the recommendations outlined in^32^; concentrations were adjusted to suit our instrumentation (Tecan Infinite® 200 PRO series plate reader, Tecan Deutschland GmbH, Crailsheim, Germany) using 96 well plates. Transparent flat bottom plates were used for absorbance measurements.

CI (NADH:decylubiquinone oxidoreductase): Permeabilized mitochondria were added to two reaction mixtures (25 mM KH_2_PO_4_, 2.5 g/l BSA, 0.3 mM NaCN, 0.1 µM myxothiazol, pH 7.2) with or without 1 µM rotenone. The final volume is double that of the sample added. After 10 min of temperature equilibration at 37 °C, 120 µM decylubiquinone was added. Reaction was started with 100 µM NADH. Initial NADH oxidation was measured at 340 nm and reaction rate was calculated as the rotenone-sensitive NADH oxidation (*ε*_NADH_ = 6.2 mM^–1^∙cm^–1^* l* = 0.7 cm) at the initial highest signal-to-noise ratio interval.

CII (succinate:2,6-dichlorophenolindophenol oxidoreductase): The permeabilized cell suspension was added to two reaction mixtures (25 mM KH_2_PO_4_, 20 mM succinate, 0.3 mM NaCN, 0.1 µM myxothiazol, 1 µM rotenone, pH 7.2) with or without 10 mM malonate. The final volume is double that of the sample added. After 10 min, 65 µM 2,6-dichlorophenolindolphenol (DCPIP) was added. Reaction was started with 50 µM decylubiquinone. Initial DCPIP reduction was measured at 600 nm and reaction rate was calculated as the malonate-sensitive DCPIP reduction (*ε*_DCPIP_ = 19.1 mM^–1^∙cm^–1^, l = 0.7 cm).

CIII (decylubiquinol:ferricytochrome C oxidoreductase) activity was assayed similar to CII with a reaction mixture (50 mM KH_2_PO_4_, 1 g/l BSA, 0.3 mM NaCN, 1 µM rotenone, 0.1 mM EDTA, 1 mM n-dodecyl-β-d-maltoside, pH 7.2) with or without 0.1 µM myxothiazol. Without delay, 92.5 µM decylubiquinol was added. Reaction was started with 50 µM ferricytochrome C. Initial ferricytochrome C reduction was recorded at 550 nm and reaction rate was calculated as the myxothiazol-sensitive cytochrome c reduction (∆*ε*_cytc_ = 18.5 mM^–1^∙cm^–1^, l = 0.7 cm).

CIV (ferrocytochrome c oxidase) activity was assayed similar to CII with a reaction mixture (20 mM KH_2_PO_4_, 0.1 µM myxothiazol, 0.45 mM n-dodecyl-β-d-maltoside, pH 7.0) and with or without 5 mM azide. After 10 min of temperature equilibration on 37 °C, reaction was started with 50 µM ferrocytochrome C. Cytochrome C was reduced freshly on the day of the measurement with dithionite. Initial cytochrome C oxidation was recorded at 550 nm. After the measurement concentrated ferricyanide was added and the absorption was recorded. Azide-sensitive pseudo first order rate constant was calculated from the ferrocytochrome c oxidation.

CV (Fo-F1 ATPase): The permeabilized cell suspension was added to two reaction mixtures (50 mM Tris, 2 mM EGTA, 1 mM MgCl_2_, pH 8.0) with or without 50 µM oligomycin. After 5 min of temperature equilibration at 37 °C measurement was started while constant shaking. Reaction was started with 2 mM ATP. Samples were taken at 1, 2 and 3 min and the reaction was quenched with equal volume of 10 w/v% trichloroacetic acid. After centrifugation (5 min, 14,000 rpm) 25 µl supernatant was added to 155 µl colour reagent (45 parts of 13.33 mM molybdenum(VI), 0.44 mM antimony(III) in 1.33 mM tartarate; 110 parts of dimethyl sulfoxide) parallel to phosphate standards. Colour development was started by adding 20 µl 1 w/v% ascorbic acid and after 15 min the absorbance was read at 890 nm. Reaction rate was calculated as the oligomycin-sensitive ATP hydrolysis to phosphate.

### Citrate synthase

The sample was added to a reaction mixture (100 mM Tris, 0.1 mM 5,5′-Dithiobis(2-nitrobenzoic acid, DTNB), 0.1 w/v% Triton-X100, pH 8.0). After 10 min of temperature equilibration at 37 °C, 0.1 mM acetyl-CoA was added. Reaction was started with 0.25 mM oxaloacetate. Initial DTNB reduction was recorded at 412 nm and reaction rate was calculated directly; background thiolase activity was in all cases negligible (*ε*_DTNB_ = 13.6 mM^–1^∙cm^–1^, l = 0.7 cm).

### Oxygen consumption and extracellular acidification rates in cell cultures

Real-time measurements of oxygen consumption rate (OCR) and extracellular acidification rate (ECAR) were performed on a microfluorimetric XF96 Analyzer (Seahorse Bioscience, North Billerica, MA, USA) as previously described^33^. Cells were seeded 1 day before addition of the inhibitors (or vehicle) in Seahorse XF96 cell culture microplates at ~ 2×10^4^ cells/well density, in growth media. At 0 h growth media were changed and the inhibitors (or vehicle) were added in assay media containing (in mM): 120 NaCl, 3.5 KCl, 1.3 CaCl_2_, 1.0 MgCl_2_, 20 HEPES, 10 glucose, 4 glutamine, pH 7.0 and the experiments were conducted at 24, 48 and 72 h. OCR and ECAR values were calculated by the XF96 Analyzer software. During the measurement, 20–26 µl of testing agents prepared in assay media were then injected into each well to reach the desired final working concentration.

### Cell viability and total cell number

HepG2 or MCF-7 cells were cultured in poly-d-Lysine-coated Corning or Greiner 96-well High Content Imaging Glass Bottom Microplates (#No. 4580, Corning Inc., USA or #No. 655892, Greiner Bio-One GmbH, Germany) for 1–2 days, at a density of approximately 2×10^4^ cells/well. Cultures were incubated in a standard CO_2_ incubator at 37 °C in assay medium containing (in mM): 120 NaCl, 3.5 KCl, 1.3 CaCl_2_, 1.0 MgCl_2_, 20 HEPES, 10 glucose, 4 glutamine at pH 7.4 and moved into the microscope for further imaging at room temperature and atmospheric CO_2_ concentration at the indicated time-points. For representative images and nucleus-based quantification of total, necrotic and apoptotic cells, nuclear staining with 1 µg/ml Hoechst 33342 dye (Invitrogen,Thermo Fisher Scientific, Corp., USA staining all cells), propidium iodide (PI) dye (0.1 µg/ml, Invitrogen, staining necrotic cells) or pSIVA-IANBD probe (polarity-Sensitive Indicator of Viability & Apoptosis- N,N′-dimethyl-N-(iodoacetyl)-N′-(7-nitrobenz-2-oxa-1,3-diazol-4-yl)ethylenediamine, Bio-Rad Laboratories Inc., USA, staining cells with exposed phosphatidylserine residues, a marker of apoptosis) were added to assay media for 20 min before image acquisition. Cell cultures were imaged with an ImageXpress Micro Confocal High Content Imaging System (Molecular Devices). Filter pairs of 377/50 and 447/60 nm for Hoechst 33342, 562/40 and 624/40 nm for propidium iodide and 475/34 and 536/40 nm were used for pSIVA-IANBD. Imaging of one 96-well plate took approximately 40 min which the cells seemed to tolerate reasonably well. During image acquisition, nine 20 × magnified fields of view (each covering 0.7209 mm^2^) were captured from each well and at least four parallel wells were imaged for each condition. Images were quantified with MetaXpress High Content Image Acquisition & Analysis Software to determine the number of objects of interest based on intensity above local background as well as minimal and maximal object sizes. The cell scoring workflow used attempted to separate touching/overlapping suprathreshold objects as well, thus the total object count was used for further quantitative characterization of the cultures. Detection and quantification of total, necrotic or apoptotic cells was performed by the MetaXpress Analysis software built-in cell scoring workflow of the blue channel, blue vs. red and blue vs. green channel images. Total number of double (PI and pSIVA) positive cells was determined by scoring workflow of the red vs. green channel images. Nuclear condensation of apoptotic cells was identified from Hoechst-positive areas visible in ImageXpress images obtained with a 20 × objective and 4 × 4 binning and quantified using a morphometric measurement pipeline in Image Analyst MKII (Image Analyst Software, Novato, CA).

### Simultaneous measurement of mitochondrial (ΔΨm) and plasma membrane potential (PMP)

HepG2 or MCF-7 cultures were grown for imaging on poly-D-Lysine-coated 8-well LabTek II chambered coverglasses (Nunc, Rochester, NY, USA) for 1–2 days, at a density of approximately 3×10^4^ cells/well. Cultures were incubated at 37 °C in imaging medium containing (in mM): 120 NaCl, 3.5 KCl, 1.3 CaCl_2_, 1.0 MgCl_2_, 20 HEPES, 10 glucose, 4 glutamine, pH 7.4 with TMRM (180 nM) plus the bis-oxonol type plasma membrane potential indicator, DIBAC_4_(3) (250 nM, Life Technologies Inc.) for 60 min before the experiment. Experiments were performed at 34 °C on an Olympus IX81 inverted microscope equipped with a UAPO 20 × 0.75 NA lens, a Bioprecision-2 xy-stage (Ludl Electronic Products Ltd., Hawthorne, NY) and a 75W xenon arc lamp (Lambda LS, Sutter Instruments, Novato, CA). Time lapses of 1342 × 1024 pixels frames (digitized at 12 bit with 4 × 4 binning, 250 ms exposure time for TMRM and 100 ms exposure time for DIBAC_4_(3) were acquired by an ORCA-ER2 cooled digital CCD camera (Hamamatsu Photonics, Hamamatsu, Japan) under control of MetaMorph 6.0 software (Molecular Devices; Sunnyvale, CA, USA). For the illumination of DIBAC_4_(3) a 490/10 nm exciter, a 505LP dichroic mirror and 535/25 emission filter were used, for TMRM a 535/20 nm exciter, a 555LP dichroic mirror and a 630/75 emitter were used, all from Chroma Technology Corp., (Bellows Falls, VT, USA). The cross-talk of TMRM and DIBAC_4_(3) emissions was eliminated by a linear spectral un-mixing algorithm implemented in Image Analyst MKII as previously described^34^, using decomposition algorithms developed in^35^. The algorithm calculates un-mixed fluorescence intensities by solving a linear equation with the cross-talk coefficient matrix. Coefficient matrix was determined by loading the cultures either with TMRM or DIBAC_4_(3) while both emissions (TMRM and DIBAC_4_(3)) were recorded. At the end of each experiment, full mitochondrial depolarization and plasma membrane depolarization was achieved by the application of mitochondrial (MDC) and plasma membrane depolarization cocktails (CDC). MDC contained (in μM): 1 valinomycin, 1 SF 6847, 2 oligomycin; CDC contained (in μM): 1 valinomycin, 1 SF 6847, 2 oligomycin, 10 nigericin, 10 monensin.

### Hypoxia treatment of cells

Plated cells were placed in the interior of a hypoxia incubator chamber (STEMCELL technologies) and flushed with 95% Nitrogen (purity: 99,99,999%) and 5% CO_2_ for 4 min, at 1.4 Bar. Subsequently, the chamber was hermetically closed and placed within a thermocontrolled incubator, at 37 °C. After 40 min, the process was repeated once and the cells were returned to the thermocontrolled incubator. Cells were processed at 24, or 48 or 72 h after this hypoxic treatment.

### Verification of hypoxia

For evaluating the extent of hypoxia in cells, cultures were treated with EF5 (2-(2-Nitro-1H-imidazol-1-yl)-N-(2,2,3,3,3-pentafluoropropyl) acetamide), a compound that binds to hypoxic cells and forms adducts^36^. The adducts were detected by a mouse monoclonal antibody (clone ELK3-51) conjugated to Alexa 488. The fluorescence of Alexa 488 was visualized by epifluorescence microscopy.

Animals: Mice were of mixed 129 Sv and C57BL/6 background. The animals used in our study were of either sex and between 2 and 6 months of age. Data obtained from liver mitochondria of mice of a particular gender or age (2, 4, or 6 months) did not yield any qualitative differences, thus all data were pooled. Mice were housed in a room maintained at 20–22 °C on a 12-h light–dark cycle with food and water available ad libitum. The study was approved by the Animal Care and Use Committee of the Semmelweis University (Egyetemi Állatkísérleti Bizottság, protocol code F16-00177 [A5753-01]; date of approval: May 15, 2017). All methods reported are in accordance with the ARRIVE guidelines (https://arriveguidelines.org).

### Isolation of mouse liver mitochondria

Liver mitochondria were isolated from mice as described in^37^.

### Determination of membrane potential (ΔΨm) in isolated mitochondria

ΔΨm of isolated mitochondria (1 mg of mouse liver mitochondria in two ml buffer medium, the composition of which is described in^38^) was estimated fluorimetrically with 5 µM rhodamine 123^38^. Fluorescence was recorded using an Oroboros O2k (Oroboros Instruments, Innsbruck, Austria) equipped with the O2k-Fluo LED2-Module with optical sensors including an LED (465 nm; < 505 nm short-pass excitation filter), a photodiode, and specific optical filters (> 560 nm long-pass emission filter)^39^. The experiments were performed at 37 °C.

### Determination of respiration in isolated mitochondria

Oxygen consumption was performed polarographically using an Oroboros O2k simultaneous to determination of ΔΨm, thus conditions were identical. The oxygen concentration (µM) and oxygen flux (pmol·s^–1^·mg^–1^; negative time derivative of oxygen concentration, divided by mitochondrial mass per volume and corrected for instrumental background oxygen flux arising from oxygen consumption of the oxygen sensor and back-diffusion into the chamber) were recorded using DatLab software (Versions 7.3.0.3 Oroboros Instruments, Innsbruck, Austria).

### Protein determination

The protein equivalent concentration was determined using the bicinchoninic acid assay and calibrated using bovine serum standards^40^ using a Tecan Infinite® 200 PRO series plate reader (Tecan Deutschland GmbH, Crailsheim, Germany).

### Lactate determination

Lactate released in the suspension was determined as described in^41^ with minor modifications: 40 µl media sample was added to 110 µl reaction mixture (final concentrations: 2.5 mM NAD^+^, 20 µM phenazine ethosulfate, 0.6 mM iodonitrotetrazolium chloride, 33.3 mM Tris base, 0.145 U bovine heart lactate dehydrogenase Lot: SLBH3714V). Lactate was measured as a difference in the formation of the coloured formazan product in 30 min with and without lactate dehydrogenase. Reaction was stopped by adding 50 µl 2 w/v% trichloroacetic acid and absorbance was measured at 525 nm. Absorbance was sub-linearly proportional to lactate concentration, but sample sensitivity did not reliably conform to that of a standard calibration, therefore results are reported in raw absorbance change per mg protein. Protein was measured after solubilisation with 0.1 M KOH.

### Reagents

Standard laboratory chemicals and digitonin were from Sigma. SF 6847 was from Biomol (BIOMOL GmbH, Hamburg, Germany). Fetal bovine serum was from Atlanta Biologicals (Lawrenceville, GA, USA) and all other tissue culture reagents were purchased from Life technologies.

### Statistics

Data are presented as mean ± SEM or ± SD as stated in the legend; significant differences between two groups of data were evaluated by Student’s *t* test, with p < 0.05 considered significant. Significant differences between three or more groups of data were evaluated by one-way analysis of variance followed by Tukey’s or Dunnett's post-hoc analysis, with p < 0.05 considered statistically significant. If normality test failed, ANOVA on Ranks was performed.

## Results

### Respiratory chain inhibitors confer a complete and persistent inhibition of their targets throughout the experimental time frame

**Figure 1:**
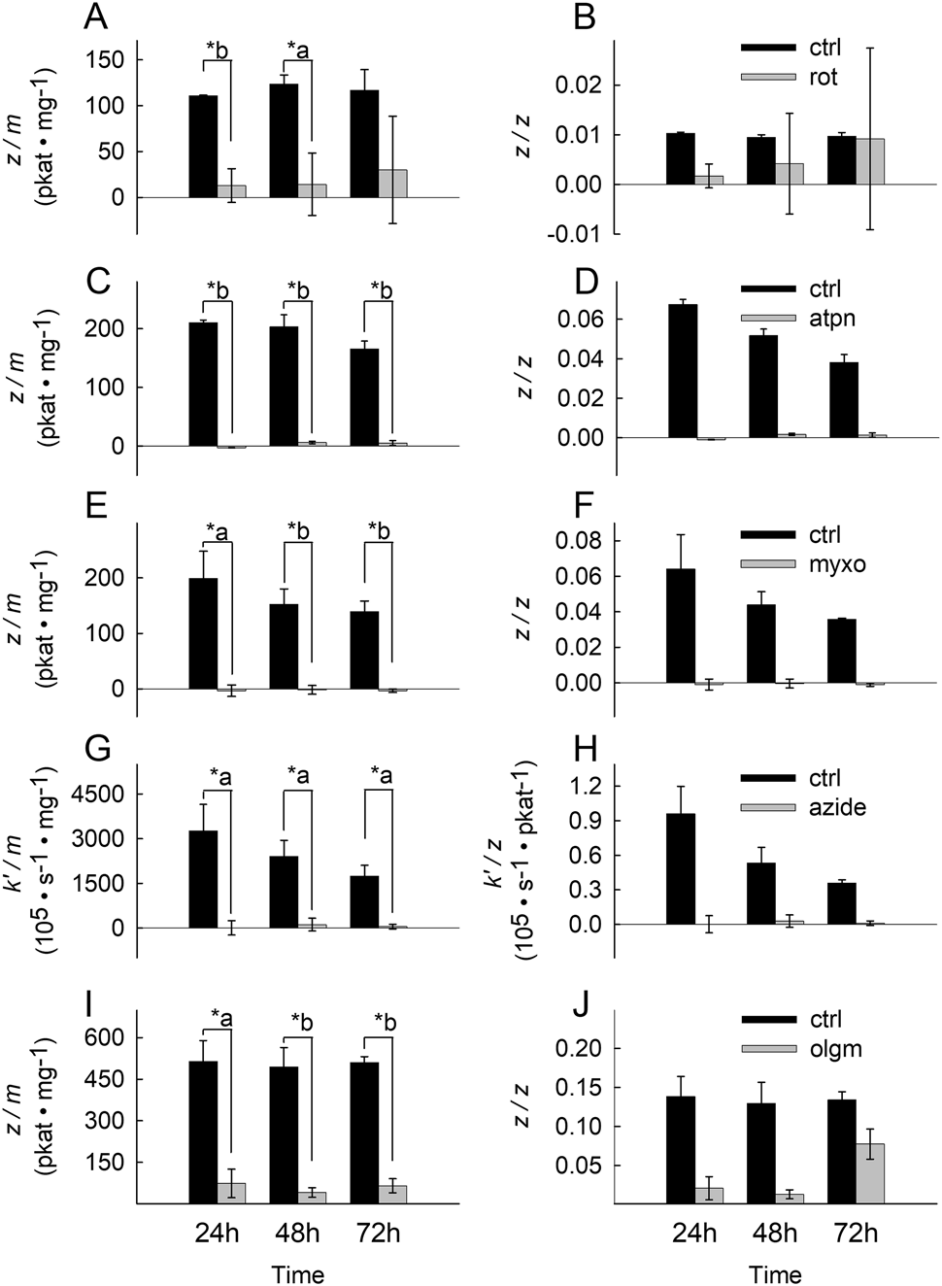
Effect of site-specific inhibitors on OXPHOS component activities in HepG2 cells over time. Bars depicting CI, CII, CIII, CIV and CV inhibitor-sensitive catalytic activities normalized to protein equivalent (pkat/mg) (**A**,**C**,**E**,**G**,**I**) or citrate synthase (CS) activity (unitless) (**B**,**D**,**F**,**H**,**J**) after incubation for 24 h, 48 h and 72 h at 37 °C under 5% CO_2_ atmosphere in assay media containing the respective RC inhibitor. The inhibitors (5 µM rotenone for CI, 1 µM atpenin A5 for CII, 1 µM myxothiazol for CIII, 1 mM azide for CIV, 5 µM oligomycin for CV) sustained complete or near complete inhibition of the respective complex for the entire duration of the experiment (grey bars), compared to untreated cells (black bars). Data indicate the mean of three biological replicates and error bars indicate 1 standard deviation. *a: p < 0.05; *b: p < 0.001.

**Figure 2:**
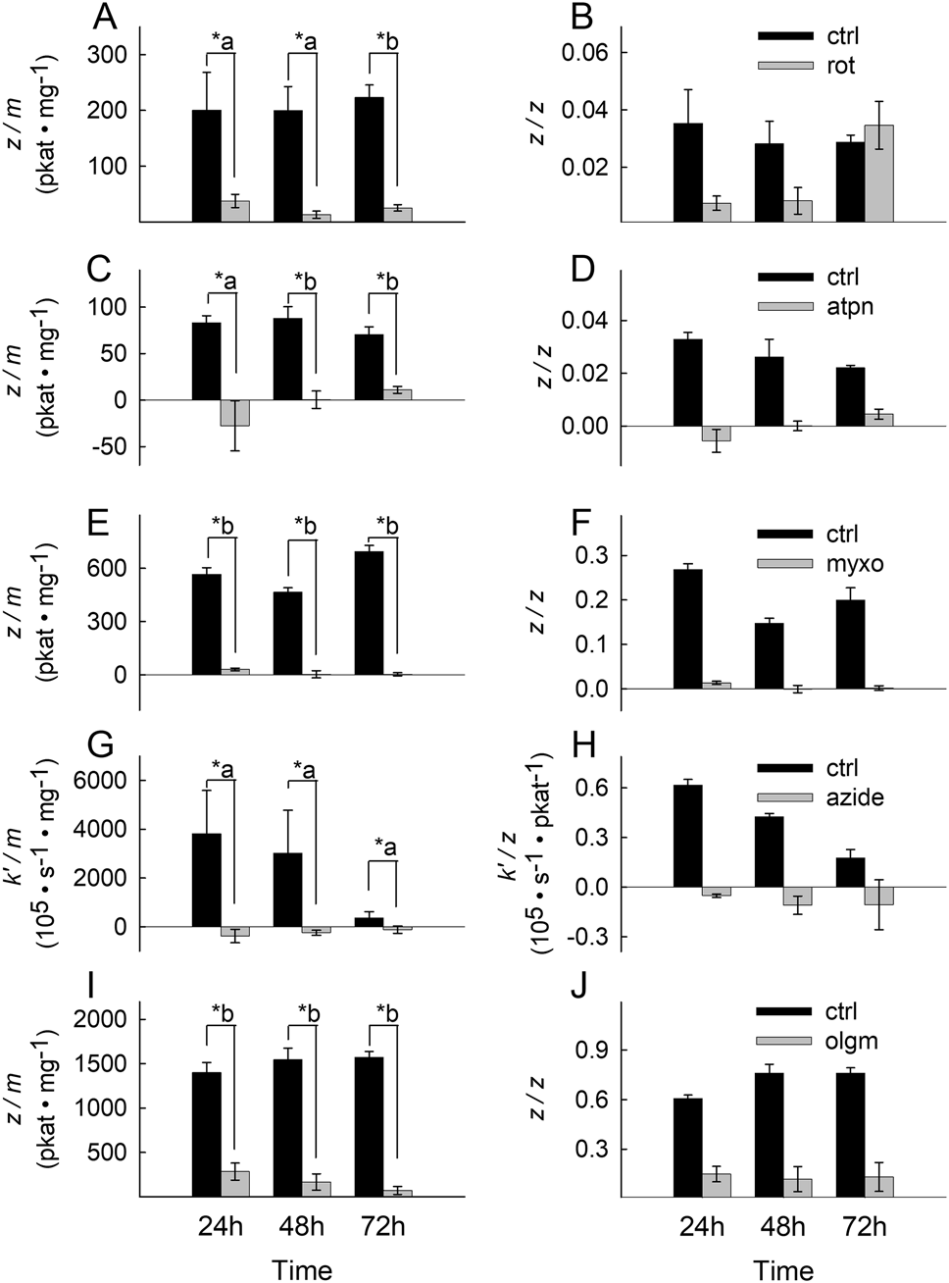
Effect of site-specific inhibitors on OXPHOS component activities in MCF-7 cells over time. Bars depicting CI, CII, CIII, CIV and CV inhibitor-sensitive catalytic activities normalized to protein equivalent (pkat/mg) (**A**,**C**,**E**,**G**,**I**) or citrate synthase (CS) activity (unitless) (**B**,**D**,**F**,**H**,**J**) after incubation for 24 h, 48 h and 72 h at 37 °C under 5% CO_2_ atmosphere in assay media containing the respective RC inhibitor. The inhibitors (5 µM rotenone for CI, 1 µM atpenin A5 for CII, 1 µM myxothiazol for CIII, 1 mM azide for CIV, 5 µM oligomycin for CV) sustained complete or near complete inhibition of the respective complex for the entire duration of the experiment (grey bars), compared to untreated cells (black bars). Data indicate the mean of three biological replicates and error bars indicate 1 standard deviation. *a: p < 0.05; *b: p < 0.001.

A main aim of the present study is to investigate the effects of site-specific RC inhibition on HepG2 and MCF-7 cells viability in a temporal manner. For this, it must be ensured that RC inhibition by the drugs is complete and sustained throughout the experiments, i.e. respiratory complex inhibition is not overcome by cellular proliferation, biotransformation and/or decomposition of the inhibitor(s) within the 72 h time frame. As shown in Fig. 1 for HepG2 cells and Fig. 2 for MCF-7 cells, CI, CII, CIII and CIV activities normalized to protein (A, C, E, G) or citrate synthase (CS) activity (B, D, F, H) the inhibitors (rotenone for CI, atpenin A5 for CII, myxothiazol for CIII and azide for CIV) sustained complete inhibition of the respective complexes for the entire duration of the experiment (grey bars), compared to untreated cells (black bars). An exception to this statement is the effect of CII inhibitor atpenin A5 on MCF-7 cells after incubation for 3 days (Fig. 2C,D); there, CII activities seem to rebound by 20–30% after experiencing complete abolition in the first 2 days. Due to the crudeness of the homogenates the specificity of the activity assay was limited to around 40–50% for CI and 60–70% for CV. In addition, the decrease in protein equivalent or citrate synthase (CS) activity over time tended to magnify the variance and error. From the data shown in Figs. 1A–H and 2A–G) it is also evident that the activities of most complexes (except CIII in MCF-7 cells shown in Fig. 2H) decreases over time, in the absence of the inhibitors. This latter phenomenon was not investigated further.

Of note, we added azide instead of the most commonly used cyanide for inhibiting CIV. The reason for this is because we discovered that on neutral pH cyanide evaporates significantly over the course of several hours, thus its potency in inhibiting CIV is decreasing, at least within the experimental time frame. Azide did not suffer from the same limitation; on the other hand, we also discovered that azide exhibits uncoupling properties in higher doses, as shown in Supplementary Fig. 1. So we opted for a 1 mM concentration as a compromise between uncoupling and inhibition.

The activity of CV (F_o_-F_1_ ATPase) is given as the oligomycin-sensitive ATP hydrolysis rate; as shown in Fig. 1I,J (for HepG2 cells) and Fig. 2I,J (for MCF-7 cells) CV activity normalized to protein or CS activity is evident in both cell lines. From the above data we conclude that both cell lines exhibit CI-CV activities and that the CI-CV inhibitors sustain inhibition of the intended complex throughout the experimental time frame.

### RC inhibitors maintain abolition of oxygen consumption rates within the experimental time frame

Having established that all drugs sustain inhibition of the respective complexes within the experimental time frame, we sought to establish the effect of complexes inhibition on in situ mitochondrial bioenergetic competence. For this, we first evaluated the effects of the inhibitors on oxygen consumption rates (OCR) and extracellular acidification rates (ECAR) in intact, adhering cells in 2D cultures. The inhibitors were added at 0 h and the experiments were conducted at 24, 48 and 72 h. Regarding CI, we used three different inhibitors: rotenone (ROT), piericidin A (PIER) and pyridaben (PRDBN). The reason for this is because rotenone has been described to inhibit microtubule assembly^42–46^, which in turn may exert an effect of cell adherence^47^; if cells detach from the plates that would lead to the result of showing less oxygen consumption from the vicinity of the plate bottom where adherent cells are expected to reside.

**Figure 3:**
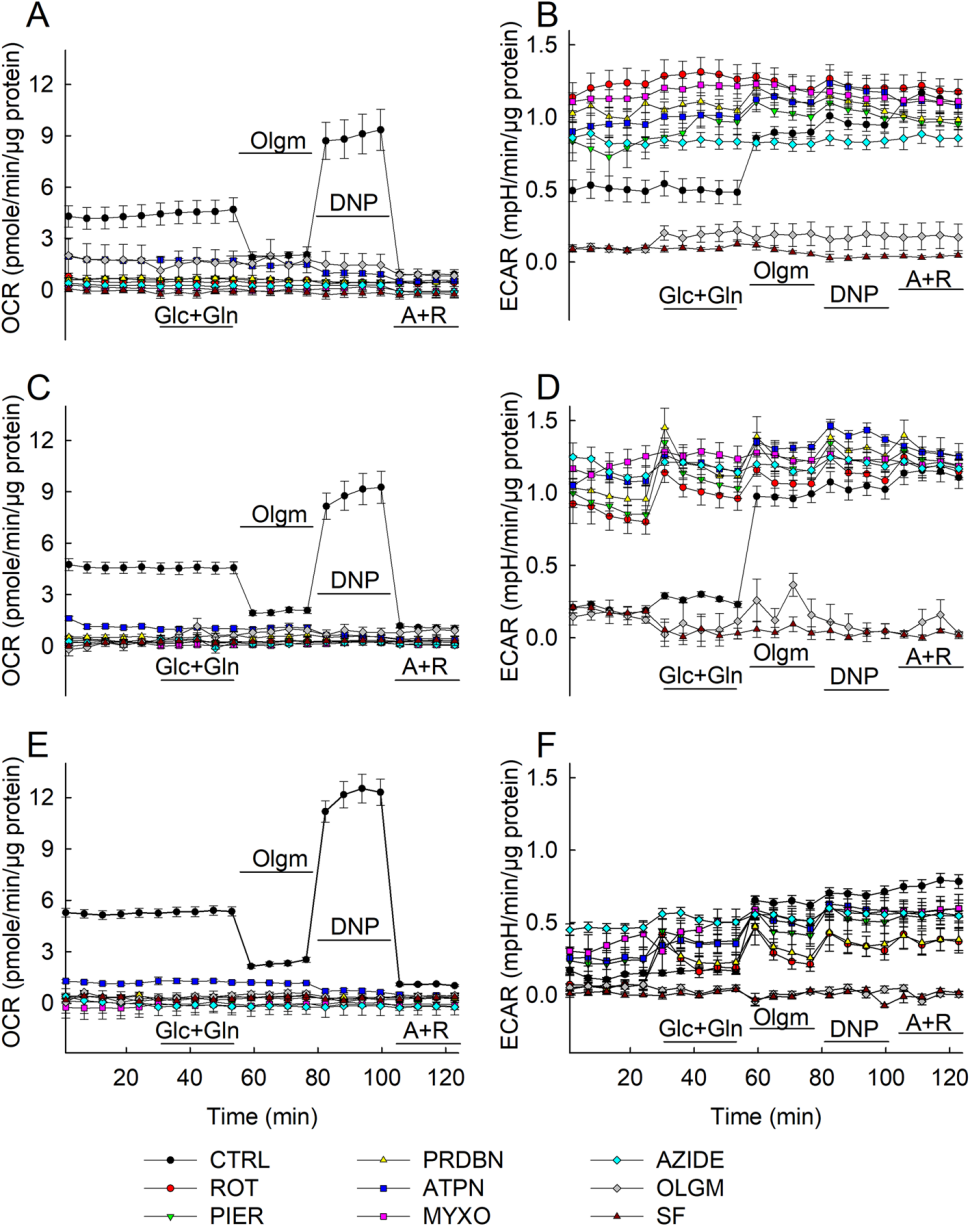
Metabolic profiling and effects of RC inhibitor additions on mitochondrial and glycolytic activities in HepG2 cells. HepG2 cells were cultured in XF96-well cell culture microplates (Seahorse Bioscience) at a density of 2 × 10^4^ cells/per well and then incubated for 24 h (**A**,**B**), 48 h (**C**,**D**) and 72 h (**E**,**F**) at 37 °C under 5% CO_2_ atmosphere in assay media containing RC inhibitors at the following concentrations: rotenone 5 µM (ROT), piericidin 1 µM (PIER), pyridaben 1 µM (PRDBN), atpenin 1 µM (ATPN), myxothiazol 1 µM (MYXO), azide 1 mM (AZIDE), oligomycin 5 µM (OLGM) and SF 6847 1 µM (SF). OCR (**A**,**C**,**E**) and ECAR (**B**,**D**,**F**) values were normalized to µgr protein content/well in the assay. Data are representative of at least three independent experiments, each additions with 12 statistical replicates and error bars indicate SEM. Glc + Gln: glucose + glutamine, A + R: antimycin and rotenone.

As shown in Fig. 3A,C and E for HepG2 cells, each and every RC inhibitor included in the media for the entire duration of the experiments (i) diminished basal OCR, (ii) abolished the oligomycin (olgm)-induced decrease in OCR and (iii) abolished the uncoupler (by DNP)-induced increase in OCR. Thus, all RC inhibitors prevented completely intact in situ mitochondria from consuming oxygen. Because each and every RC inhibitor abolished OCR, this means that OCR was almost exclusively mitochondrial; the validity of this statement is only limited by the detection threshold of OCR, i.e. the Seahorse 96 analyzer. This is also in line with the finding that addition of antimycin and rotenone (A + R) shown in the end of every Seahorse experiment decreased OCR to very low (but not zero) levels. However, it is surprising that inhibition of CII also led to complete abolition of OCR; one may have expected that CII blockade would have let the CI→ CIII→ CIV electron flow unaffected, but this was not the case. On the other hand, as expected, combined inhibition of CI + CV or CIII + CV led to nearly complete collapse of ∆ψm, shown in Supplementary Fig. 2. In line with the OCR data, ECAR experiments performed in the same HepG2 cells at the same time shown in Fig. 3B,D and F at 24, 48 and 72 h respectively demonstrate that (i) each and every RC inhibitor present in the media led to an increase in ECAR, and (ii) where no RC inhibitor was present, addition of oligomycin led to a rebound increase in ECAR, reaching levels as with the 24–72 h long treatment with RC inhibitors. The results support the conclusion that RC inhibitors abolished OXPHOS that led to compensatory increases in glycolytic fluxes yielding pyruvate and lactate formation, acidifying the media. The lack of effect of adding glucose and glutamine (indicated in the panels) on OCR and ECAR attests to the fact that cells were not starved by either substrate.

**Figure 4:**
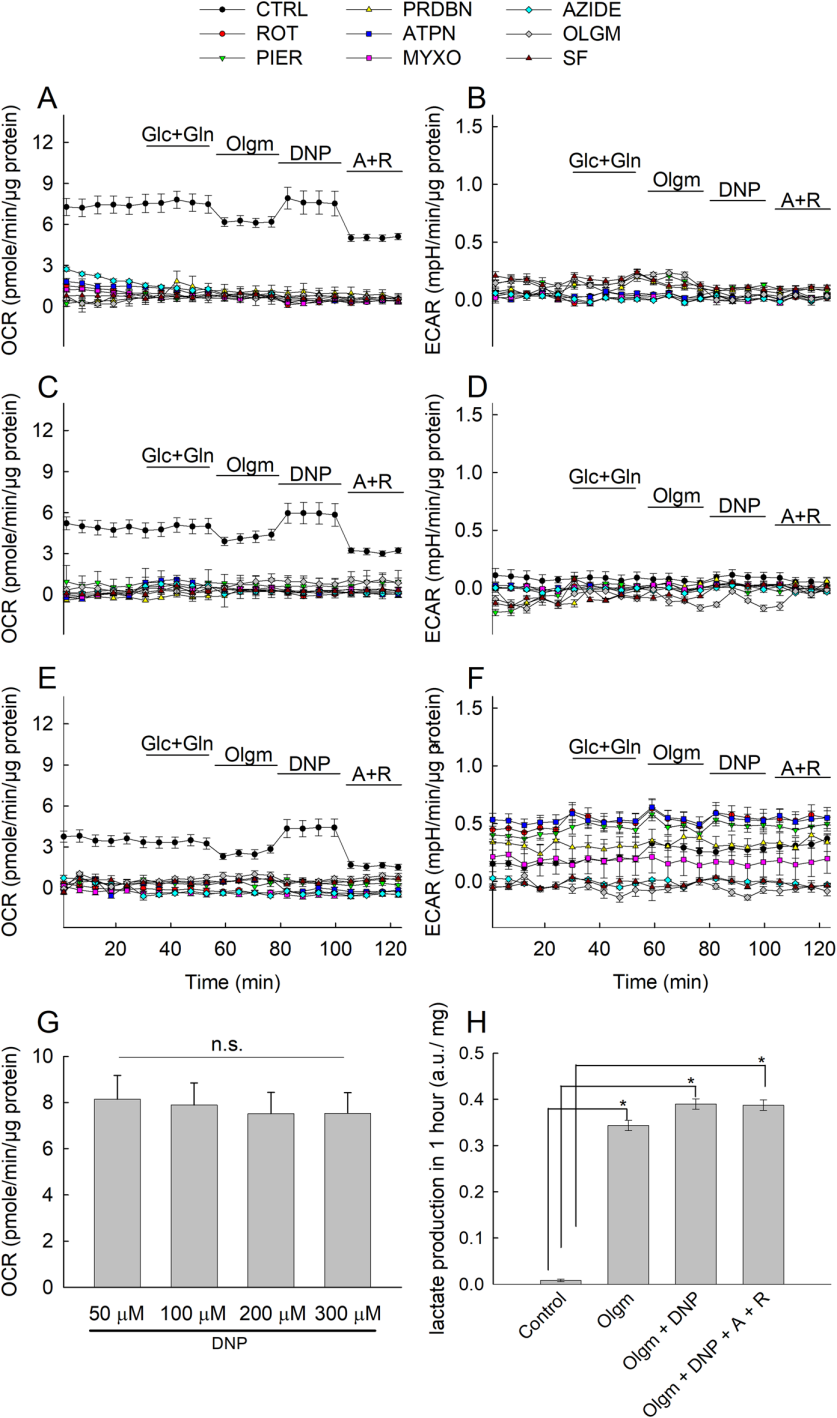
Metabolic profiling and effects of RC inhibitor additions on mitochondrial and glycolytic activities in MCF-7 cells. MCF-7 cells were cultured in XF96-well cell culture microplates (Seahorse Bioscience) at a density of 2 × 10^4^ cells/per well and then incubated for 24 h (**A**,**B**), 48 h (**C**,**D**) and 72 h (**E**,**F**) at 37 °C under 5% CO2 atmosphere in assay media containing RC inhibitors. Concentrations of RC inhibitors were identical to those indicated in the legend of Fig. 3. OCR (**A**,**C**,**E**) and ECAR (**B**, **D**,**F**) values were normalized to µgr protein content/well in the assay. Data are representative of at least three independent experiments, each addition with 12 statistical replicates and error bars indicate SEM. (**G**) Dose-dependent effect of uncoupler 2,4-DNP (DNP) additions on mitochondrial OCR in MCF-7 cells. The concentrations indicated imply total concentration of the uncoupler. OCR values were normalized to µgr protein content/well in the assay. Data are representative of three independent experiments, with 32 statistical replicates and error bars indicate SEM. (**H**) Lactate release in MCF-7 cells. 3∙10^5^ MCF-7 cells were cultured for 1 day in 6-well plates followed by 1 day of incubation in an assay media, the composition of which was: 120 mM NaCl, 3.5 mM KCl, 20 mM HEPES, 1.3 mM CaCl_2_, 1 mM MgCl_2_, 10 mM glucose, 4 mM glutamine, pH 7.4. Lactate concentration difference induced by the addition of 2 µM oligomycin, 100 µM 2,4-dinitrophenol (DNP), 1 µM antimycin and 1 µM rotenone in 1 h was measured as indicated under materials and methods (n = 5). Unit is raw absorbance per mg protein. *p < 0.001.

OCR of MCF-7 cells was much less than that of HepG2, in accordance to most other reports (see Introduction). Modest oligomycin-induced decreases followed by DNP-induced increases in OCR (in the absence of other RC inhibitors) is observed in Fig. 4A,C and E (black symbols). The presence of each and every RC inhibitor abolished all these responses. The results support the conclusion that our MCF-7 sub-lineal cultures exhibit poor OCR indicating very weak OXPHOS. Furthermore, it is noteworthy that addition of antimycin and rotenone (A + R) to MCF-7 cells did not yield near zero OCR levels while also being comparable to the levels recorded prior to addition of any inhibitors, thus, this pre-A + R oxygen consumption is not entirely attributed to mitochondria. Pre-treatment of cultures for 24–72 h with any RC inhibitor however, resulted in the loss of the basal OCR rates. The latter findings attest to a serious pitfall: OCR of MCF-7 cultures may not be entirely mitochondrial but 24–72 h treatment with RC inhibitors abolishes the non-mitochondrial origin of oxygen consumption. Addition of cyanide salts to the A + R cocktail was not considered, because cyanide inhibits oxygen-consuming enzymes such as oxidases and peroxidases^48^ which are unrelated to cytochrome oxidase.

Because the DNP-induced increase in OCR of MCF-7 cells was small -at least compared to the one obtained with HepG2 cells- we investigated whether the concentration of this uncoupler was suboptimal by being insufficient or too much; in the former case, it would not yield maximum respiration, and in the latter case the undesirable effect of DNP inhibiting respiration would be a confounding factor. We thus checked the effect of DNP on OCR of MCF-7 cells in a dose-dependent manner. As shown in Fig. 4G, although 50 µM led to the highest OCR value, the consecutive additions of DNP at concentrations indicated in the panel (cumulative, within 50–300 µM) conferred no statistically significant changes in OCR, compared to each other (one-way ANOVA, Tukey test). That means that the amount of DNP used (100 µM) was appropriate to elicit the maximum response attainable in MCF-7 cells.

The ECAR data obtained from MCF-7 cells (also performed in the same cells at the same time as for OCR) shown in Fig. 4B,D and F reveal very low rates in pH alterations in the media for any solid conclusions to be drawn. This may sound surprising, assuming that these cells should exhibit a glycolytic profile. To address this, we measured lactate in the extracellular space; as shown in Fig. 4H, the exact same treatments applied in OCR/ECAR Seahorse experiments (oligomycin, followed by addition of DNP, followed by addition of antimycin and rotenone but allowing 1 h interval instead of 4–20 min as for the Seahorse experiments) revealed very large emanation of lactate, implying strong glycolysis. It is possible that the amount of lactate formed with 4–20 min is not sufficient to elicit a detectable change in mpH that dictates ECAR.

From Fig. 4 it is also evident that 1–3 day pre-treatment with any RC inhibitor yields less OCR and ECAR values compared to the acute addition of A + R. To address this further, we compared citrate synthase activities as means of mitochondrial content as a function of RC inhibitor pre-treatment time. As shown in Supplementary Fig. 3, there is a time-dependent decrease of citrate synthase activity in RC inhibitor treated MCF-7 cells with the exception of atpenin, compared to the un-treated cells. This means that RC inhibitor treatment for 1–3 days diminishes mitochondrial content. We conservatively attribute this to mitophagy^49^; this phenomenon was not investigated further.

### Titration of the uncoupler SF 6847 as a function of in situ mitochondrial membrane potential (∆ψm)

Prior to testing the effect of maximum uncoupling on cell viability, we titrated the uncoupler SF 6847 and recorded TMRM fluorescence (reporting both plasma membrane and mitochondrial membrane potential) subtracted from the DIBAC_4_(3) fluorescence (reporting only plasma membrane potential) in HepG2 and MCF-7 2D cultures using wide-field epifluorescence. As shown in Supplementary Fig. 7A and B for HepG2 and MCF-7 cell cultures respectively, the uncoupler SF 6847 dose-dependently decreased the fluorescence values. When this decrease started to exhibit a plateau phase, a membrane potential-dissipating cocktail—MDC, the composition of which is detailed under materials and methods- was applied to ensure that no further loss of fluorescence could be achieved. From the experiments shown in Supplementary Fig. 7A and 7B we deduced that 1 µM SF 6847 was sufficient to confer complete uncoupling in both cell culture types.

### The effect of bioenergetic impairment on HepG2 and MCF-7 cells viability

**Figure 5:**
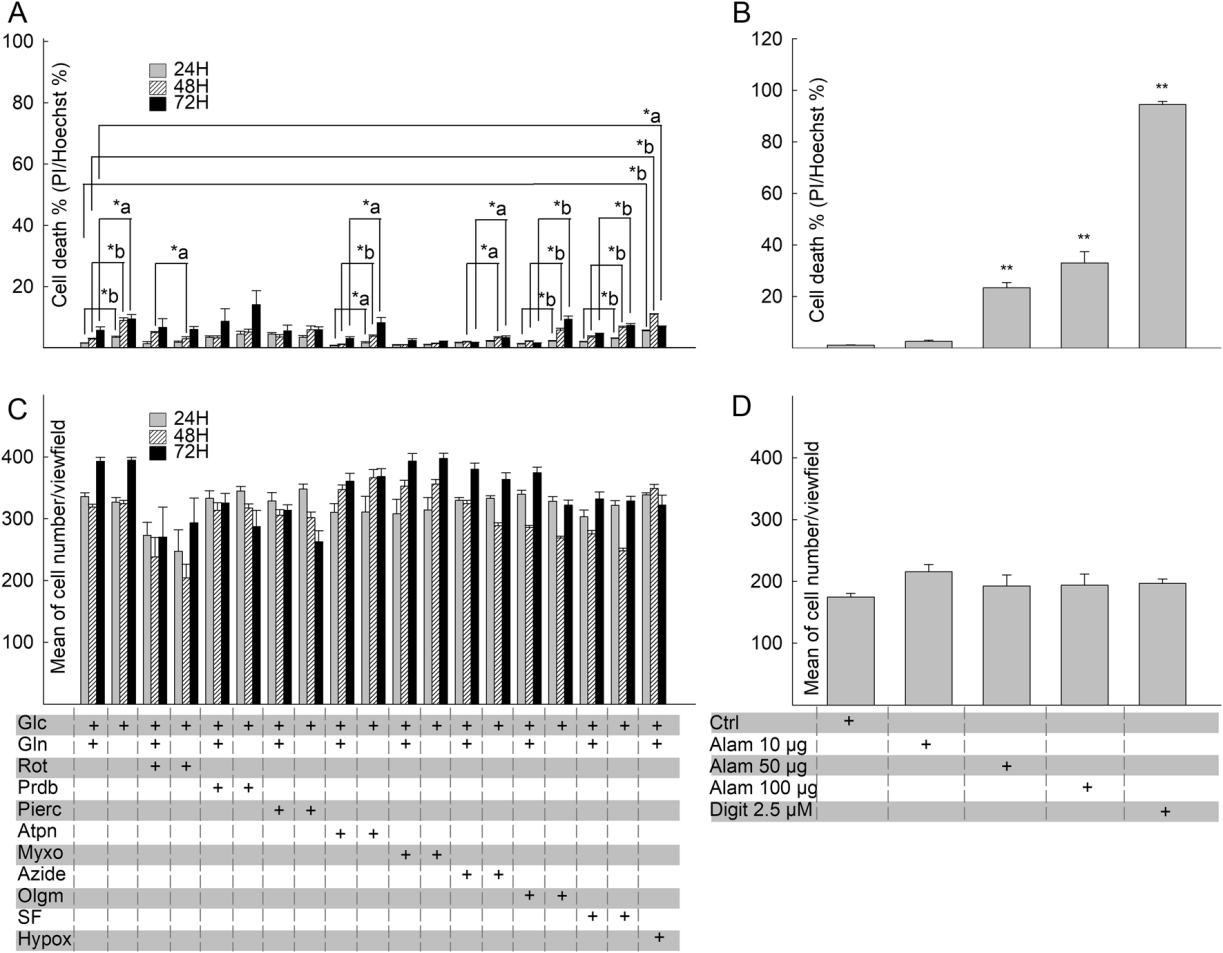
Effect of site-specific RC inhibition on the viability of HepG2 cancer cell line. (**A**) 24 h, 48 h and 72 h site-specific RC inhibition-induced cytotoxicity of HepG2 cells was assessed using nucleus-based quantification of dead (propidium iodide (PI) positive)/ total (Hoechst 33342 (Hoechst) positive) cells. (**B**) Effects of the pore-forming peptide alamethicin (Alam) and the detergent digitonin (Digit) on the viability of HepG2 cells. **p < 0.001 (one-way ANOVA, Dunnett’s test). (**C**) Mean number of HepG2 cells/field of view after 24 h, 48 h and 72 h site-specific RC inhibition. (**D**) Mean number of HepG2 cells/field of view after 15 min alamethicin or digitonin application. Bars indicate mean of at least three independent experiments, each addition with 12–32 statistical replicates and error bars indicate SEM. Concentrations of RC inhibitors were identical to those used in the metabolic profiling of HepG2 cells and see it in details in the legend of Fig. 3. *a: p < 0.05; *b: p < 0.001.

Having established that the RC inhibitors abolish the activity of respective complexes completely and eliminate OCRs throughout the experimental time frame, we next tested the effect of the inhibitors on cell viability. This variable was examined in the presence and absence of exogenously added glutamine; this is because most cancer cell lines depend on glutaminolysis supporting oxidative decarboxylation and catabolism of glutamine through the citric acid cycle and/or reductive carboxylation towards fatty acid synthesis^50^. As shown in Fig. 5A, no RC inhibitor increased the death rate of HepG2 cells, irrespective of glutamine availability. *In lieu* of chemical anoxia conferred by azide, we also tested the effect of true hypoxia by subjecting the cultures in oxygen-free environments for 24–72 h. Mindful of the technical difficulty in achieving true severe hypoxia, we performed immunohistochemical analysis of EF5 (2-(2-Nitro-1H-imidazol-1-yl)-N-(2,2,3,3,3-pentafluoropropyl) acetamide) adduct formation^36^. These adducts only form in severely hypoxic cells and can be visualized by applying a fluorophore-conjugated antibody directed against them. As shown in Supplementary Fig. 4, both HepG2 and MCF-7 cultures subjected to 24–72 h-long hypoxia led to strong labelling by the Alexa 488-conjugated antibody directed against EF5 adducts. Inclusion of EF5 in the absence of hypoxia led to virtually no labelling. Although we cannot accurately estimate the extent of hypoxia, the fact that EF5 adducts appeared to such a degree implies that oxygen concentration must have been < 0.1%. Similarly to the effect of azide, hypoxia treatment lead to a very minor increase in HepG2 cell death compared to controls, at least over the course of the experimental time frame (Fig. 5A).

To verify that our method of checking for cell viability (PI/Hoechst 33342) is valid, we tested the effects of the pore-forming peptide alamethicin and also that of the detergent digitonin. As shown in Fig. 5B, alamethicin caused a dose-dependent increase in the PI/Hoechst33342 ratio implying loss of plasma membrane integrity which commits a cell to death; digitonin also led to a nearly 100% loss of plasma membrane integrity within 15 min of treatment. RC inhibitors, alamethicin or digitonin did not exert a significant effect on total cell number, depicted in Fig. 5C and D. This affords the assurance that our data are not significantly altered by cell detachment; this is important since the method for testing cell viability was high-content automated microscopy, i.e. cells are imaged at specified time intervals.

**Figure 6:**
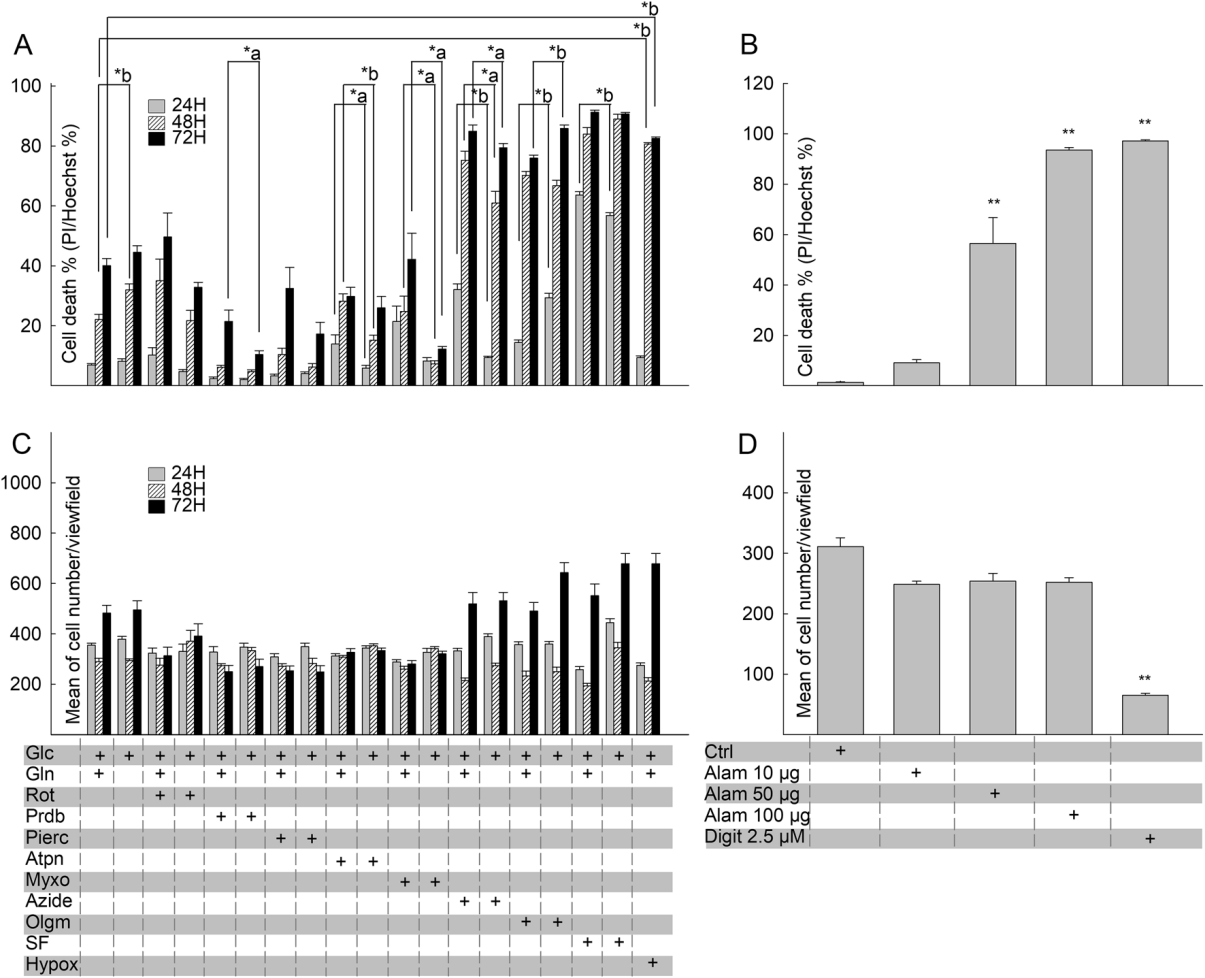
Effect of site-specific RC inhibition on the viability of MCF-7 cancer cell line. (**A**) 24 h, 48 h and 72 h site-specific RC inhibition-induced cytotoxicity of MCF-7 cells was assessed using nucleus-based quantification of dead (propidium iodide (PI) positive)/total (Hoechst 33342 (Hoechst) positive) cells. (**B**) Effects of the pore-forming peptide alamethicin (Alam) and the detergent digitonin (Digit) on the viability of MCF-7 cells. **p < 0.001 (one-way ANOVA compared to control, Dunnett’s test). (**C**) Mean number of MCF-7 cells/field of view after 24 h, 48 h and 72 h site-specific RC inhibition. (**D**) Mean number of MCF-7 cells/field of view after 15 min alamethicin or digitonin application. Bars indicate mean of at least three independent experiments, each addition with 12–32 statistical replicates and error bars indicate SEM. *p < 0.001 (one-way ANOVA compared to control, Dunnett’s test) Concentrations of RC inhibitors were identical to those used in the metabolic profiling of MCF-7 cells described in the legend of Fig. 3. For all other panels: *a: p < 0.05; *b: p < 0.001.

On the other hand, MCF-7 cells exhibited considerable sensitivity to RC inhibitors, hypoxia or uncoupling; indeed, as shown in Fig. 6A, the presence of azide or oligomycin blocking CIV and CV, respectively, led to a significant elevation in cell death; in a similar manner, true hypoxia or uncoupling by SF6847 also led to a universal cell death of the cultures. To verify that the technique of assessing cell viability is also valid for MCF-7, we tested the effects of alamethicin and digitonin, just as for HepG2 cells. As shown in Fig. 6B, alamethicin caused a dose-dependent increase in cell death; digitonin led to a nearly 100% loss of cell viability. Total cell number of MCF-7 remained relatively unaffected (Fig. 6C) except in the case of digitonin, that led to a drop to ~ 25%, shown in Fig. 6D; this is probably because digitonin detaches MCF-7 cells much more readily than other cell types from the bottom of the plate.

Having established the total number of cells per condition, we also re-normalized the OCR and ECAR data, for reasons outlined in^51^; results are shown in Supplementary Figs. 5 and 6, for HepG2 and MCF-7 cell cultures, respectively.

Mindful that aspartate availability has been reported to rescue respiration-deficient cells^52–55^, we tested the effect of including an excess of aspartate (10 mM) in the media feeding MCF-7 cells, and check whether this would prevent RC-inhibitor cell death. As shown in Supplementary Fig. 8A, the inclusion of 10 mM aspartate prevented CII-, CIII- and CIV-inhibitor mediated cell death by up to 15% (in Supplementary Fig. 8B, it is shown that cell numbers remained unaffected.). Although the effect on cell death is statistically significant, we conclude that aspartate availability cannot be the sole factor in regulating RC inhibitor-mediated MCF-7 cell death.

### The effect of bioenergetic impairment on apoptotic markers in HepG2 and MCF-7 cells

**Figure 7:**
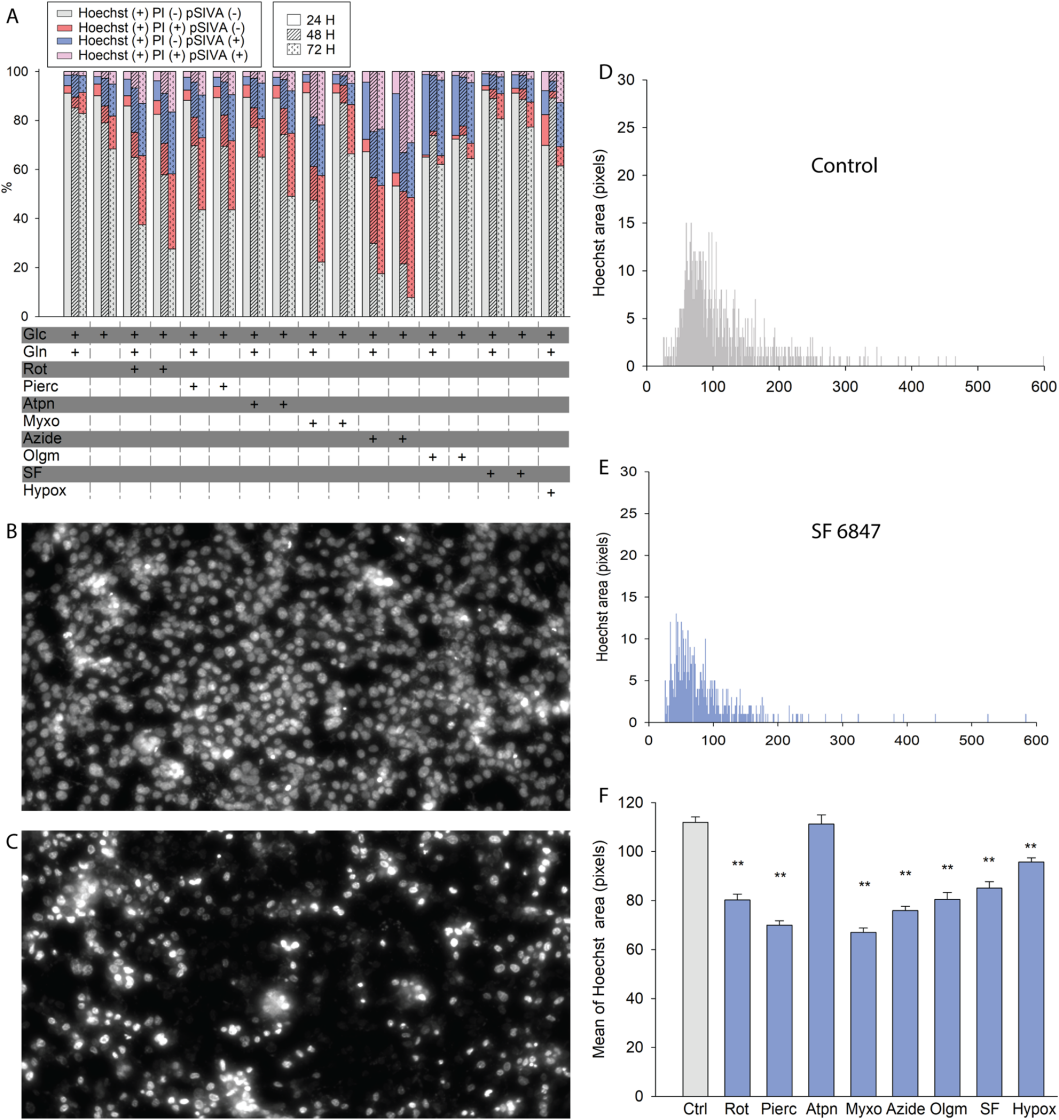
Effect of site-specific RC inhibition on apoptotic (and necrotic) markers in HepG2 cancer cells. (**A**) 24 h, 48 h and 72 h site-specific RC inhibition-induced cytotoxicity of HepG2 cells was assessed using nucleus-based quantification of dead (propidium iodide (PI) positive)/ total (Hoechst 33342 (Hoechst) positive) and pSIVA positive (apoptotic) cells. Statistics are shown in supplementary table 1. (**B**) representative image of HepG2 cells stained with Hoechst prior to any RC inhibitor treatment. (**C**) representative image of HepG2 cells stained with Hoechst treated with SF 6847 for 72 h. (**D**) quantification of Hoechst area (pixels) of all images as the one shown in panel B of control HepG2 cells. (**E**) quantification of Hoechst area (pixels) of all images as the one shown in panel C, treated with SF 6847 for 72 h. (**F**) quantification of all mean Hoechst areas obtained from all treatments for HepG2 cell cultures. **p < 0.001 compared to control (one-way ANOVA compared to control, Dunnett’s test).

**Figure 8:**
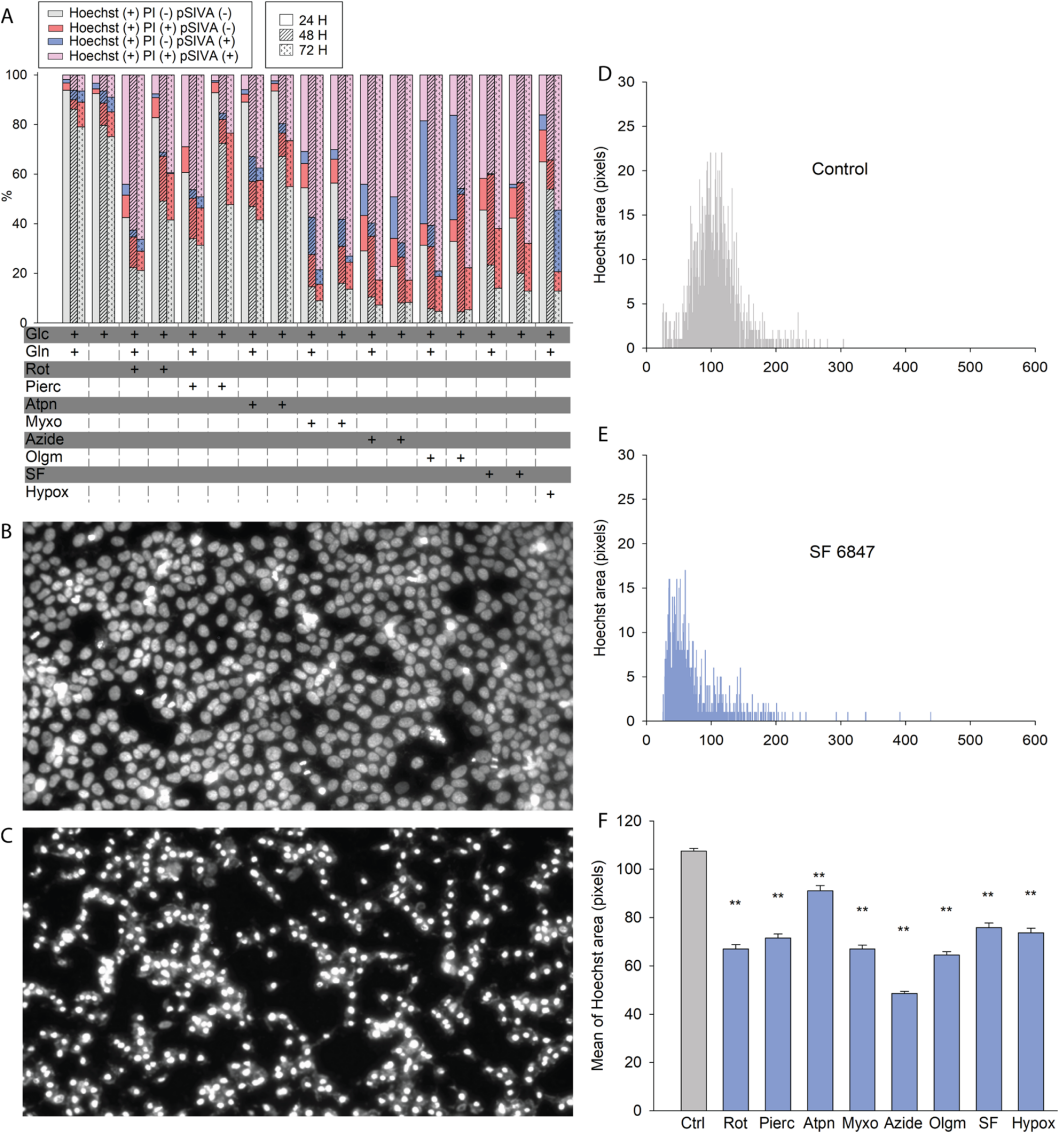
Effect of site-specific RC inhibition on apoptotic (and necrotic) markers in MCF-7 cancer cells. (**A**) 24 h, 48 h and 72 h site-specific RC inhibition-induced cytotoxicity of MCF-7 cells was assessed using nucleus-based quantification of dead (propidium iodide (PI) positive)/total (Hoechst 33342 (Hoechst) positive) and pSIVA positive (apoptotic) cells. Statistics are shown in Supplementary Table 2. (**B**) representative image of MCF-7 cells stained with Hoechst prior to any RC inhibitor treatment. (**C**) representative image of MCF-7 cells stained with Hoechst treated with SF 6847 for 72 h. (**D**) quantification of Hoechst area (pixels) of all images as the one shown in panel B of control MCF-7 cells. (**E**) quantification of Hoechst area (pixels) of all images as the one shown in panel C, treated with SF 6847 for 72 h. (**F**) quantification of all mean Hoechst areas obtained from all treatments for MCF-7 cell cultures. **p < 0.001 compared to control (one-way ANOVA, Dunnett’s test).

In the above experiments, cell viability test was synonymous to loss of plasma membrane integrity, quantified by the PI/Hoechst 33342 ratio which is a necrotic marker. To address the effect of bioenergetic impairment on markers of apoptosis in which loss of plasma membrane integrity is not a prerequisite but cell death may ensue in a delayed manner, we looked for (i) exposed phosphatidylserine, an early-onset apoptotic marker and (ii) nuclear condensation (pyknosis), an intermediate-onset marker of apoptosis. Phosphatidylserine exposure was quantified by staining cells with pSIVA-IANBD probe; we performed this simultaneous to staining with PI and Hoechst 33342 and classified the Hoechst-positive cells as apoptotic only, necrotic only, or both apoptotic and necrotic. As shown in Fig. 7A for HepG2 and 8A for MCF-7, cultures were exposed to the same bioenergetic impairment treatments as before for 24–72 h, and pSIVA ( +), PI ( +)and Hoechst ( +) cells were quantified using high-content automated microscopy. For HepG2 cells it is evident that there are pSIVA ( +) PI (–) cells (blue) implying apoptosis but not necrosis, but there is also a considerable number of cells that express both markers (pink). For MCF-7 cells, it can be observed (Fig. 8A) that phosphatidylserine exposure and loss of plasma membrane integrity co-exist for a much higher percentage of cells (pink). Statistics for both panels 7A and 8A are summarized in Supplementary Tables 1 and 2, respectively.

Nuclear condensation was observed in practically all cells (except for atpenin-treated HepG2 cultures) exposed to bioenergetic impairment; representative images of Hoechst area imaging of HepG2 and MCF-7 cells before (Figs. 7B and 8B, respectively) and after (panels 7C and 8C, respectively) application of SF 6847 are depicted. By performing morphometric analysis and quantification of the Hoechst-signal areas, we obtained histograms (x-axis representing counts of individual nuclei) as shown in Fig. 7D and E (for HepG2) and Fig. 8D and E (for MCF-7) in the absence or presence of the uncoupler SF 6847. Quantification of histograms for all bioenergetic conditions are shown in Fig. 7F and Fig. 8F for HepG2 and MCF-7 cells, respectively. Histograms of Hoechst areas from each bioenergetic condition are shown in Supplementary Figs. 9 And 10 for HepG2 and MCF-7 cells, respectively.

From the above we concluded that bioenergetic impairment was associated with some extent of phosphatidylserine exposure that was observed to a certain extent with loss of plasma membrane integrity, while all cells except for atpenin-treated HepG2 cultures exhibited nuclear condensation, irrespective of whether they exhibited intact or damaged cell membranes upon RC inhibitor treatment.

## Discussion

The main question addressed in the present work was the impact of bioenergetic impairment on cancer cells viability, specifically necrosis. We have also examined the impact of RC poisons on two apoptotic markers, the early-onset phosphatidylserine exposure^56^ and the intermediate onset nuclear condensation^57^. Although we present evidence for both apoptotic markers, we caution that they do not necessarily foretell cell death: many types of cancer cells exhibit high levels of phosphatidylserine exposure that enhances their ability to resist chemotherapy-induced apoptosis and even promotes survival and metastasis^58^. Likewise, cancer cells have been reported to evade apoptotic cell death even when their nuclei have condensed^59^. Thus, although the cells that exhibited apoptotic but not necrotic markers were considered as ‘live’ within the experimental time frame of our study, we cannot exclude the possibility that these cells would die at a later time. Overall, we specifically addressed the relation of necrotic cell death to site-specific mitochondrial dysfunction. To this end, the nature of the bioenergetic impairment was exhaustively addressed by using site-specific poisons of mitochondrial functions. Hereby, drugs were used that would either impair the ability of mitochondria to (i) develop membrane potential (using an uncoupler) or (ii) maintain a protonmotive force (using CI-CIV inhibitors or severe hypoxia), or (iii) perform ATP synthesis (using a CV inhibitor). Each and every regiment aimed to prevent mitochondria from yielding ATP by OXPHOS: SF 6847 would uncouple electron transport from ATP synthesis and also abolish the ATP-yielding directionality of the F_o_-F_1_ ATP synthase, the inhibitors CI-CIV or severe hypoxia would inhibit the electron transport thus abolish the protonmotive force, while oligomycin would prevent ATP formation directly by inhibiting the F_o_-F_1_ ATP synthase. Our results unequivocally show that no bioenergetic impairment led to a decrease in viability of HepG2 cells, while these cultures exhibited strong OXPHOS activity and respiratory capacity. On the other hand, MCF-7 cultured cells viability exhibited sensitivity to some bioenergetic impairment regiments, but also showed very weak OXPHOS. This lack of correlation urges for caution when linking cancer cell survival as a function of OXPHOS, also arguing that no extrapolations can be made from one cell type or condition, to another. Relevant to this, it has been recently reported that the tumor-growth inhibitory activity of complex I poisons is not due to energy depletion; instead they mitigate cancer growth by altering the pH of the tumor microenvironment^60^. The following findings deserve further scrutiny:

(i) Why would HepG2 cells invest in expressing all respiratory chain components, be capable of OCR, ECAR, ∆ψm and yet be resistant to any bioenergetic impairment regime that would abolish mitochondrial ATP efflux? In the literature, the list of moonlighting functions by mitochondrial proteins is constantly expanding^61–63^, however, it is puzzling (at least to these authors) as to why express the components of the entire respiratory chain, be functional, and yet not need it.
(ii) By the same token, why would MCF-7 cells exhibit so little -if any-mitochondrial bioenergetic competence to the point that it is doubtful that they need OXPHOS, and yet be so sensitive to some (but not all) bioenergetic impairment regimes? To this end, it may be possible that MCF-7 are critically dependent on this meagre bioenergetic OXPHOS, and by abolishing it -no matter how little- is detrimental to them. Relevant to this, the effect of severe hypoxia or CIV inhibition but not CIII in conferring MCF-7 cell death is puzzling; in theory, CIV could be fuelled by metabolites such as glutathione or proteins (such as p66Shc), when CIII is inhibited^64^^,^^65^. Such possibilities may be worth investigating.
(iii) The residual OCR in MCF-7 cells after the acute addition of antimycin and rotenone blocking CIII and CI respectively, must be highlighted. This points to a non-mitochondrial component consuming oxygen; thus, whenever oxygen consumption is observed, it should not automatically be attributed to mitochondria, let alone OXPHOS.

Overall, our data highlight that the relation between OXPHOS and cell viability (specifically addressing necrosis) is not straightforward, and it must be examined in cell- and condition-specific manner. The importance of this is exemplified by the evolving concept that cancer could be managed by metabolic drugs^49^.

## Supplementary Information

Supplementary Figure 1.

Supplementary Figure 2.

Supplementary Figure 3.

Supplementary Figure 4.

Supplementary Figure 5.

Supplementary Figure 6.

Supplementary Figure 7.

Supplementary Figure 8.

Supplementary Figure 9.

Supplementary Figure 10.

Supplementary Legends.

Supplementary Table 1.

Supplementary Table 2.

## Data Availability

The datasets used and/or analysed during the current study available from the corresponding author (C.C.) on reasonable request.
