# Supplementary figures and images for: Viability of HepG2 and MCF-7 cells is not correlated with mitochondrial bioenergetics
Source: Sci Rep. 2023 Jul 4;13:10822. doi: 10.1038/s41598-023-37677-x (PMC10319846; doi:10.1038/s41598-023-37677-x)

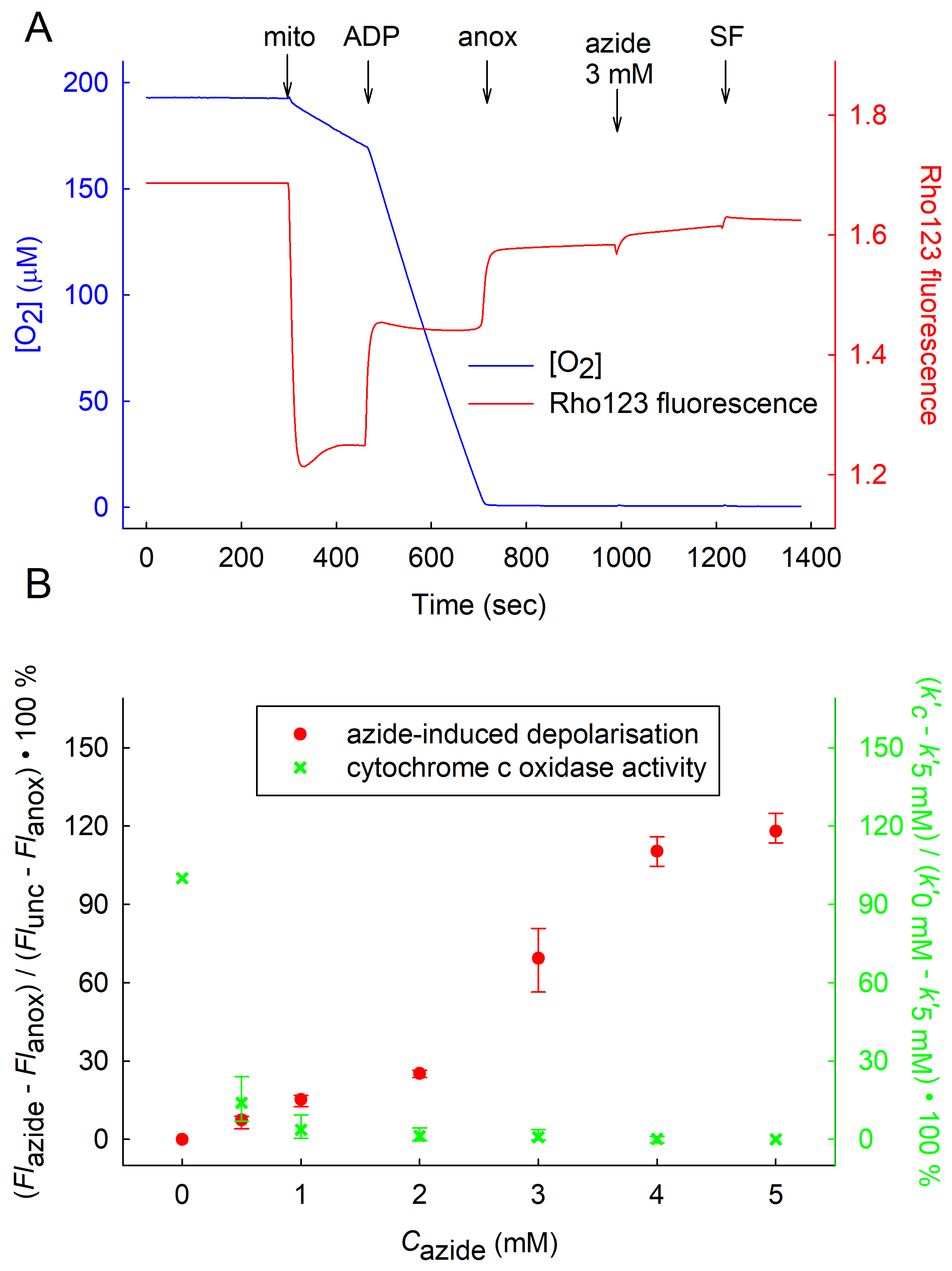

Supplement: Supplementary file 1 — Supplementary Figure 1. [file 41598_2023_37677_MOESM1_ESM.tif]

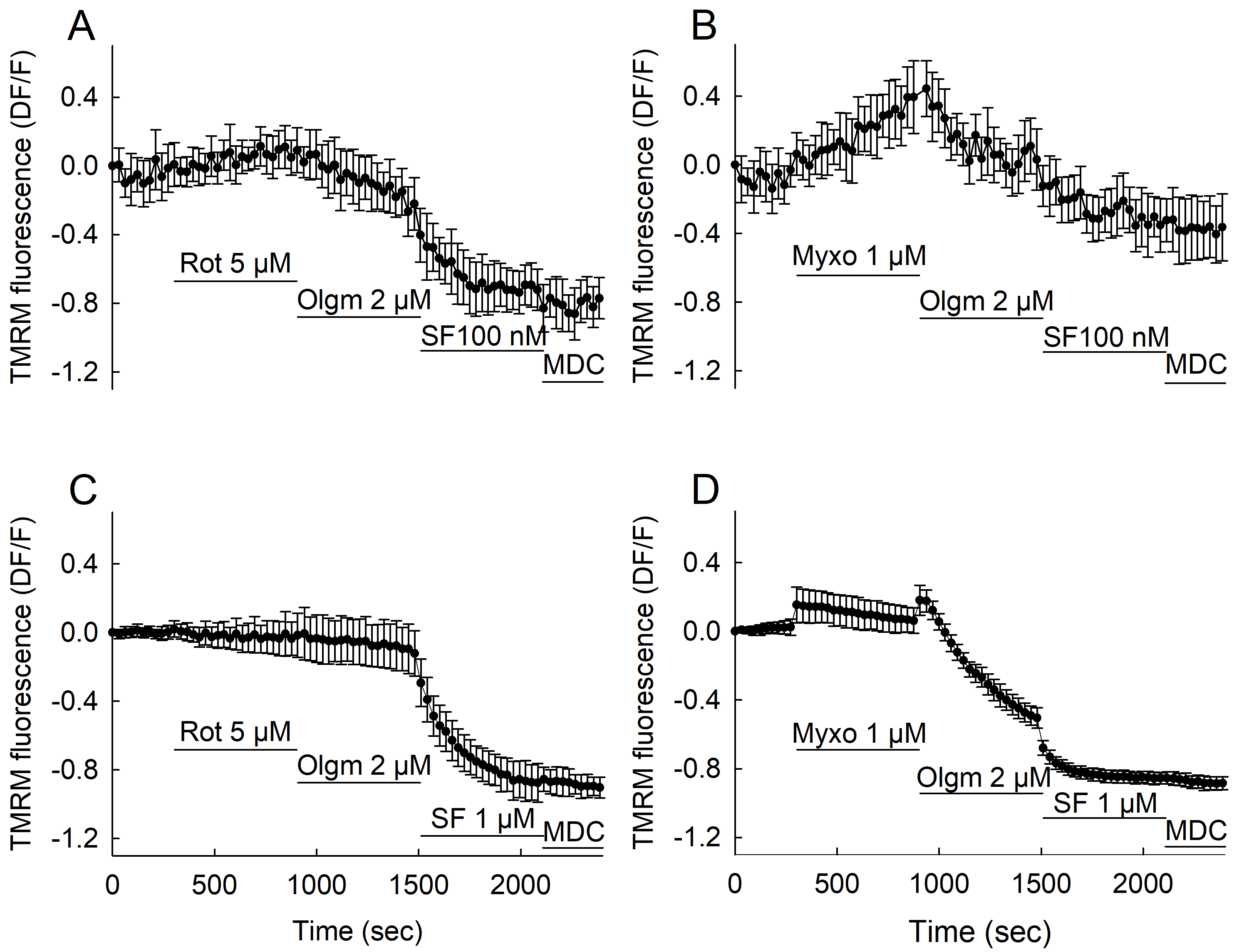

Supplement: Supplementary file 2 — Supplementary Figure 2. [file 41598_2023_37677_MOESM2_ESM.tiff]

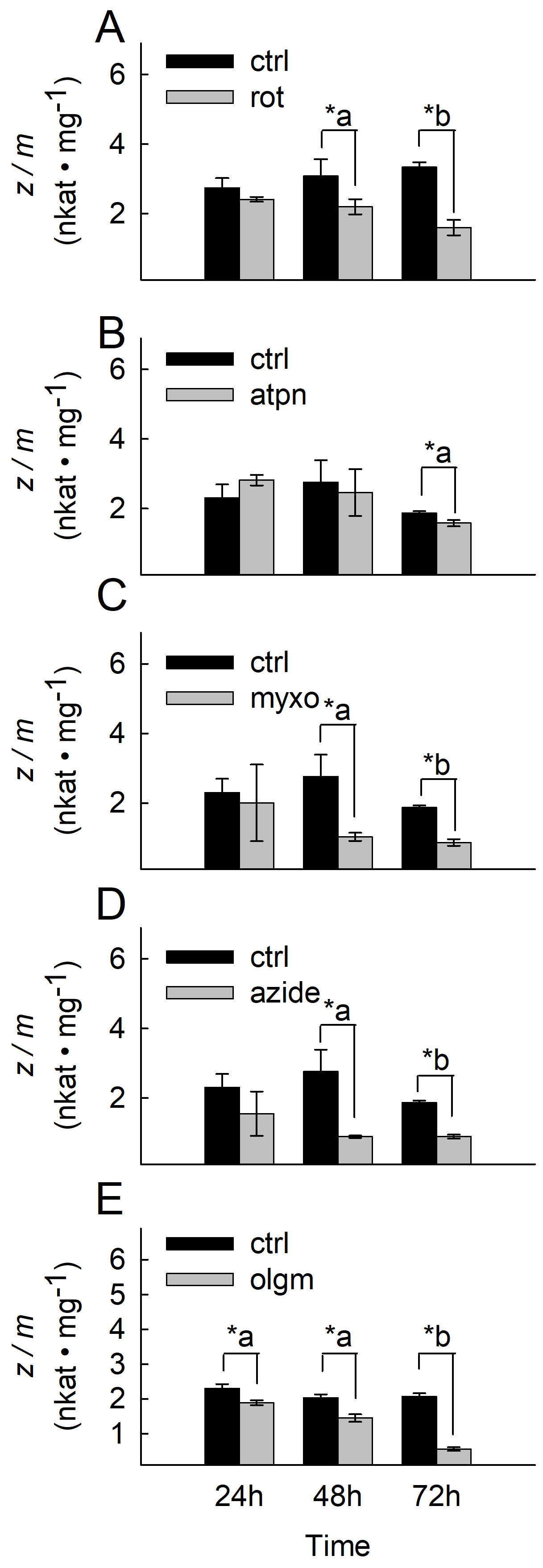

Supplement: Supplementary file 3 — Supplementary Figure 3. [file 41598_2023_37677_MOESM3_ESM.tiff]

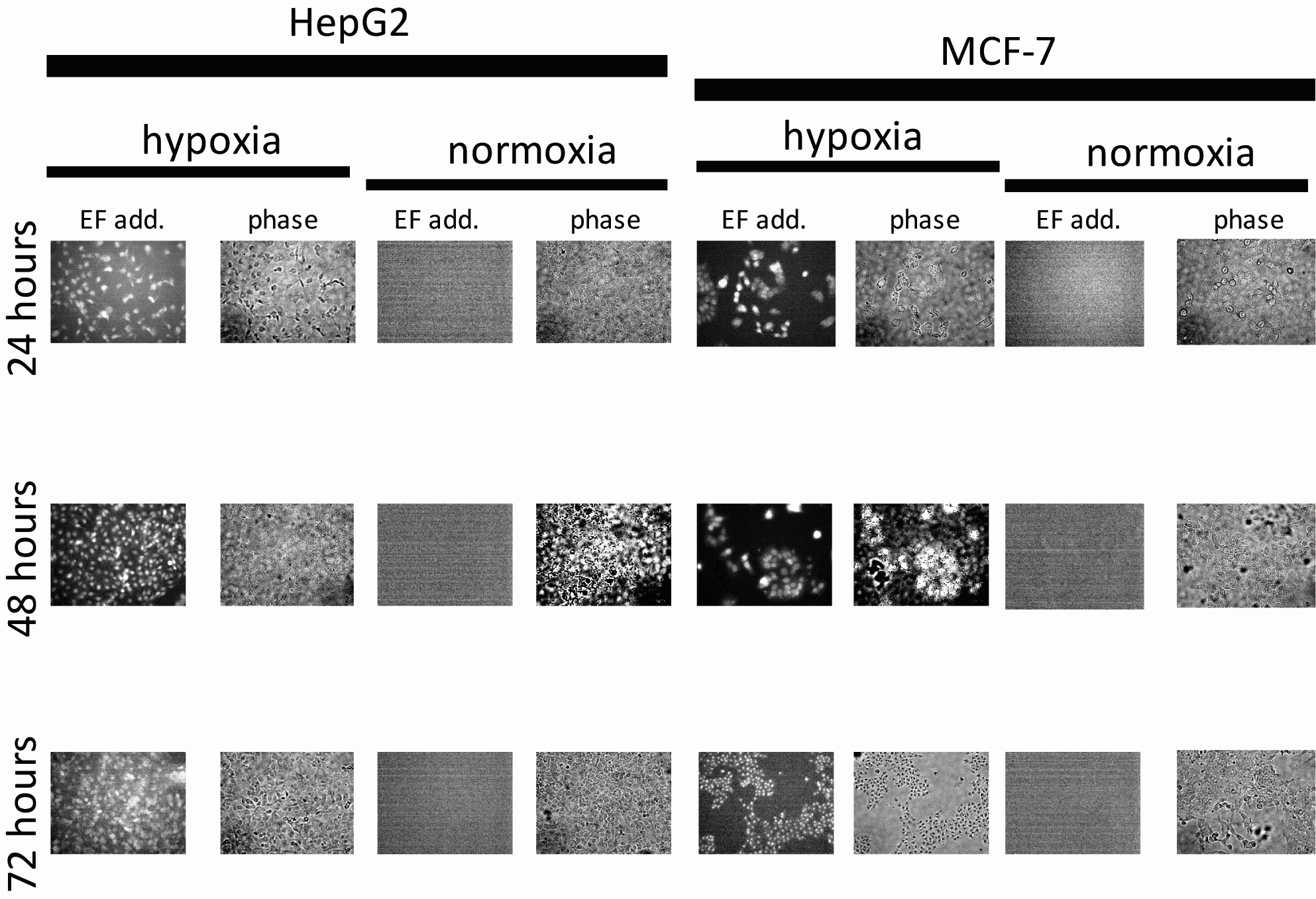

Supplement: Supplementary file 4 — Supplementary Figure 4. [file 41598_2023_37677_MOESM4_ESM.tiff]

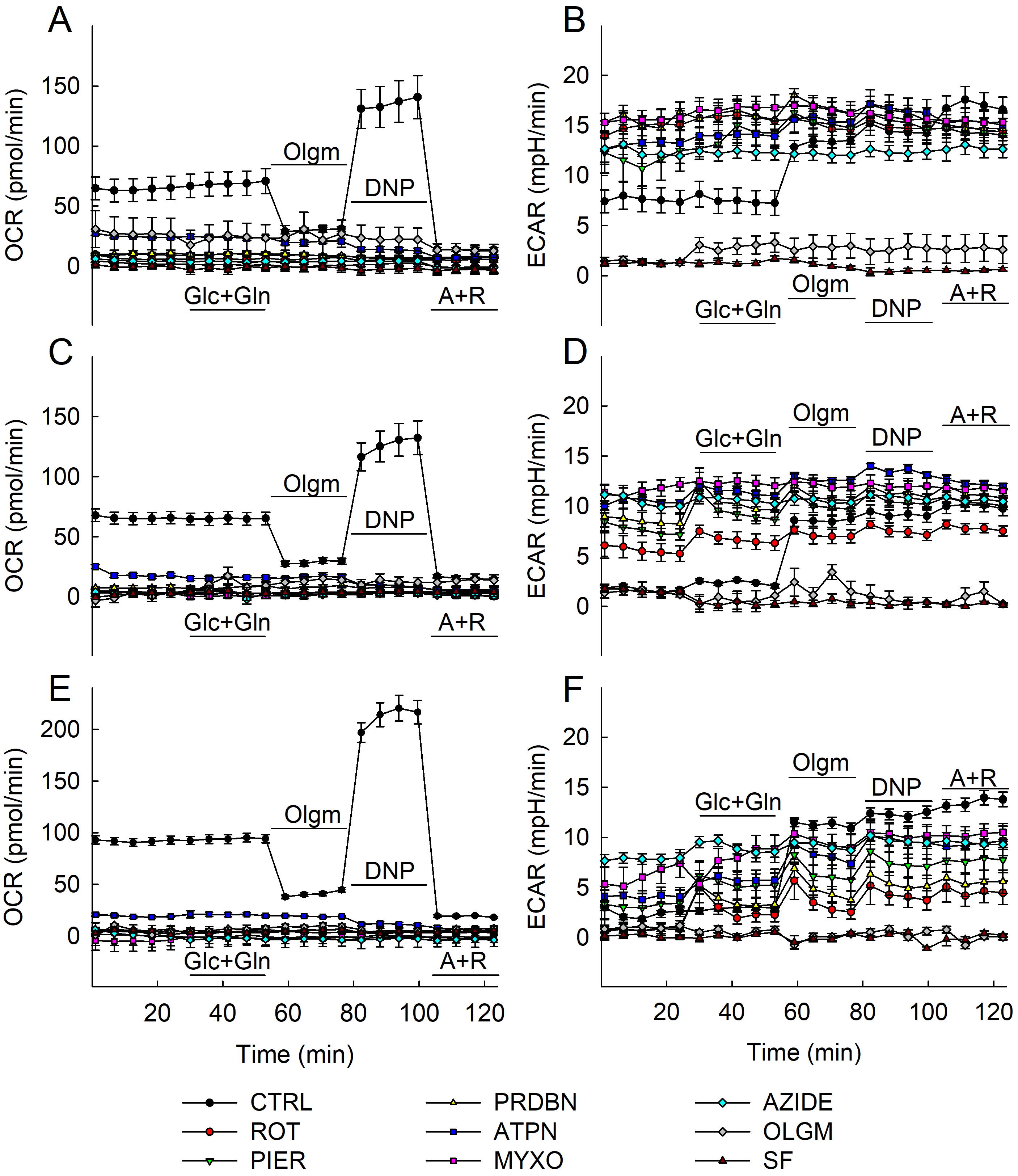

Supplement: Supplementary file 5 — Supplementary Figure 5. [file 41598_2023_37677_MOESM5_ESM.tif]

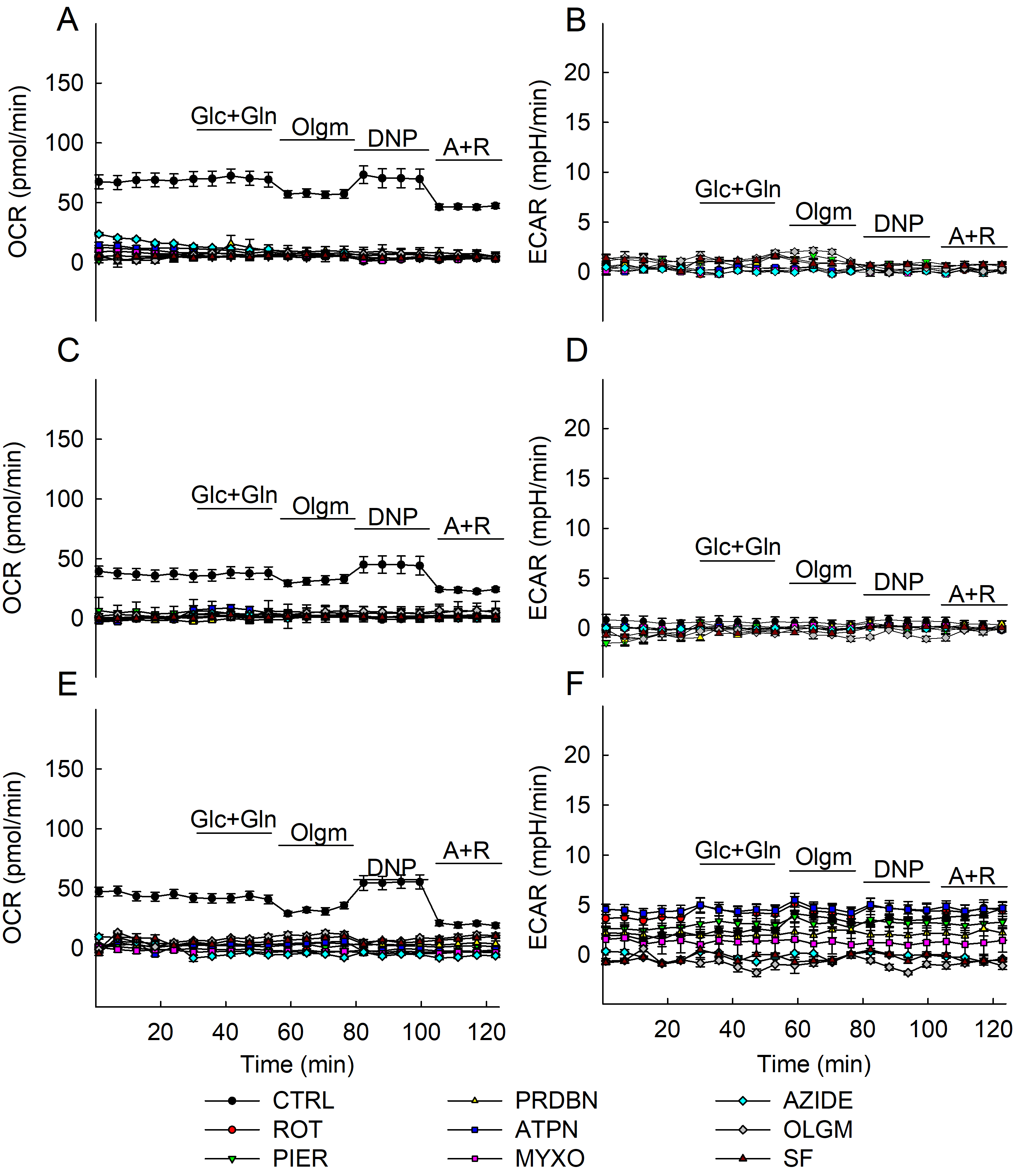

Supplement: Supplementary file 6 — Supplementary Figure 6. [file 41598_2023_37677_MOESM6_ESM.tif]

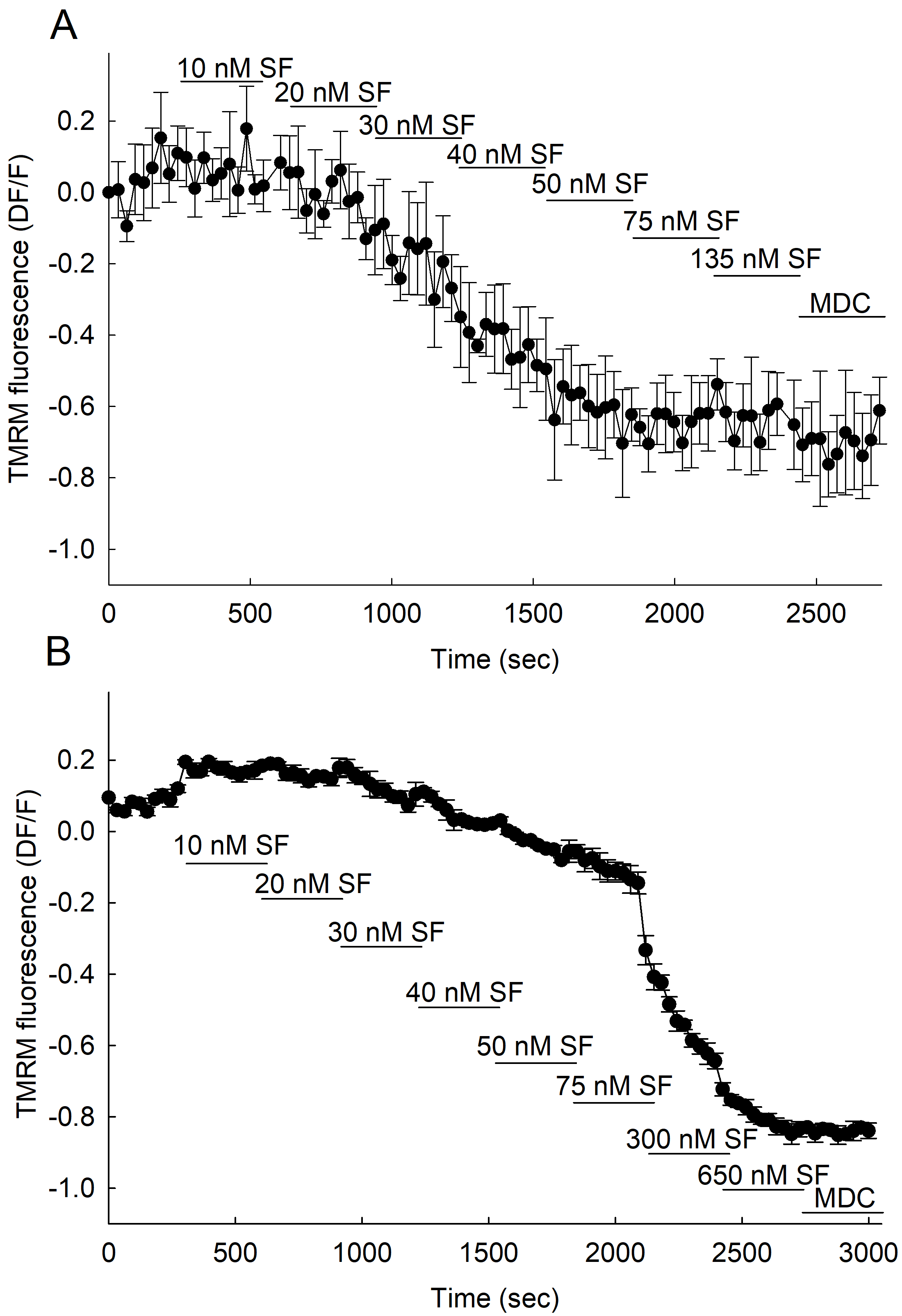

Supplement: Supplementary file 7 — Supplementary Figure 7. [file 41598_2023_37677_MOESM7_ESM.tif]

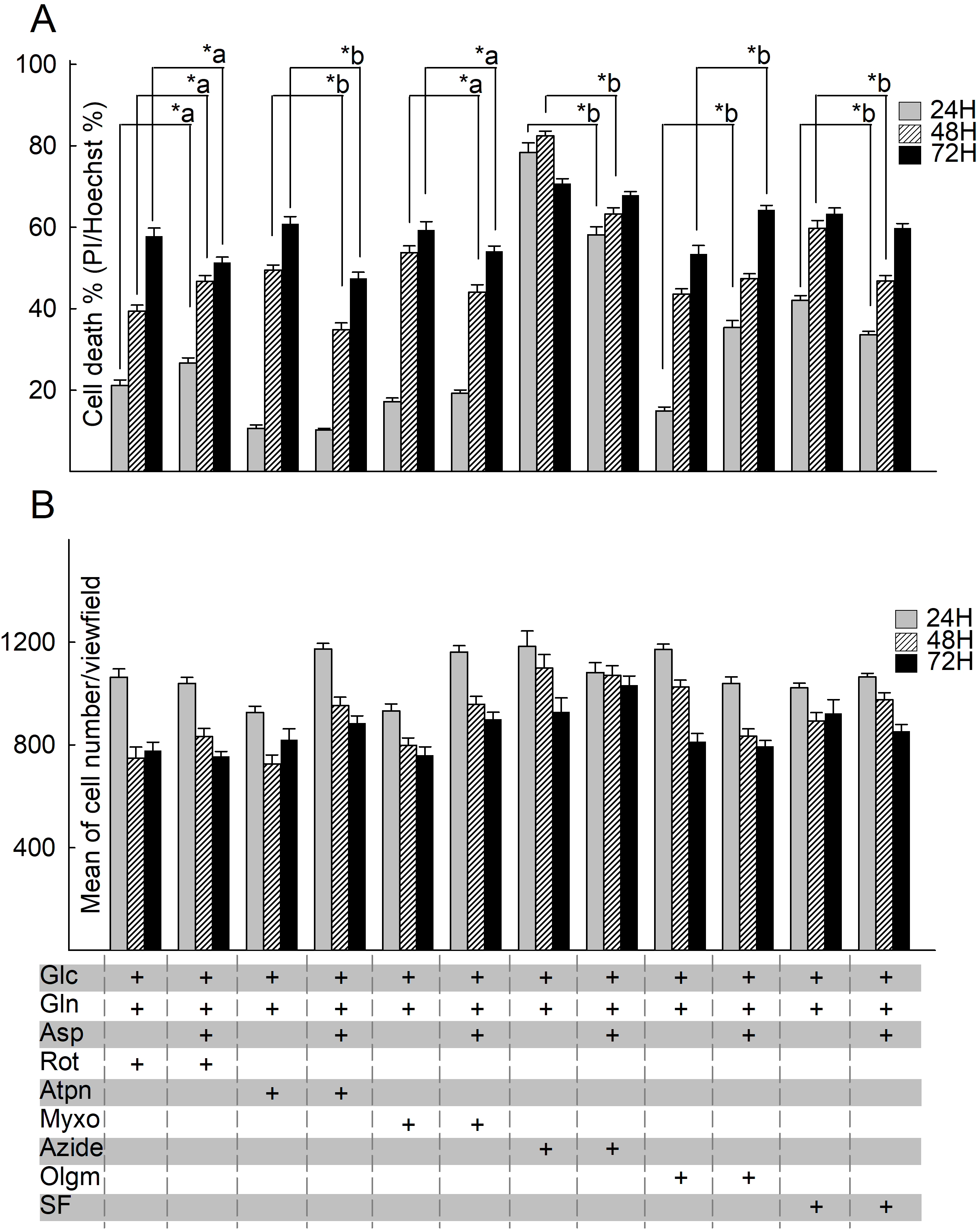

Supplement: Supplementary file 8 — Supplementary Figure 8. [file 41598_2023_37677_MOESM8_ESM.tif]

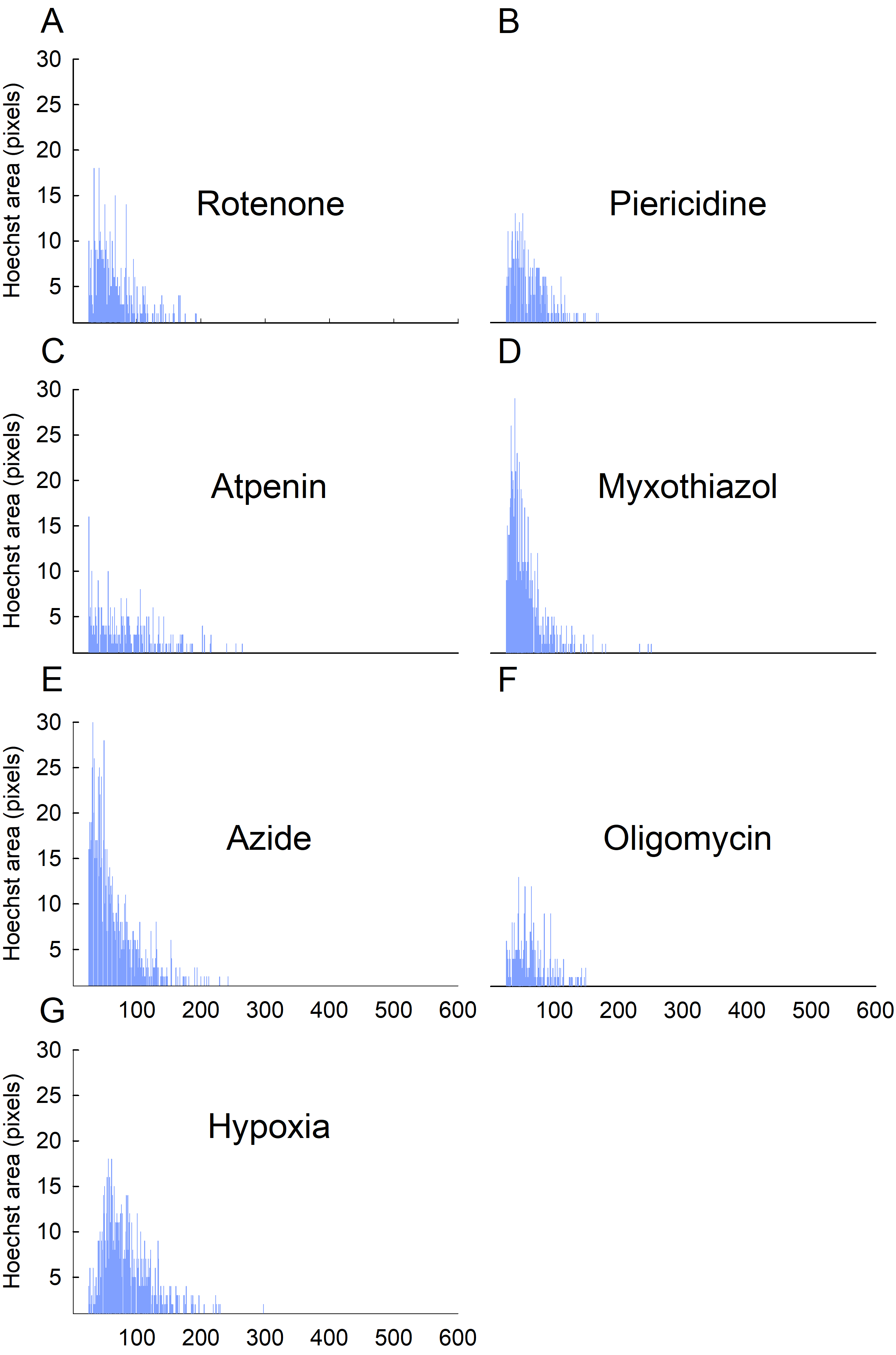

Supplement: Supplementary file 9 — Supplementary Figure 9. [file 41598_2023_37677_MOESM9_ESM.tif]

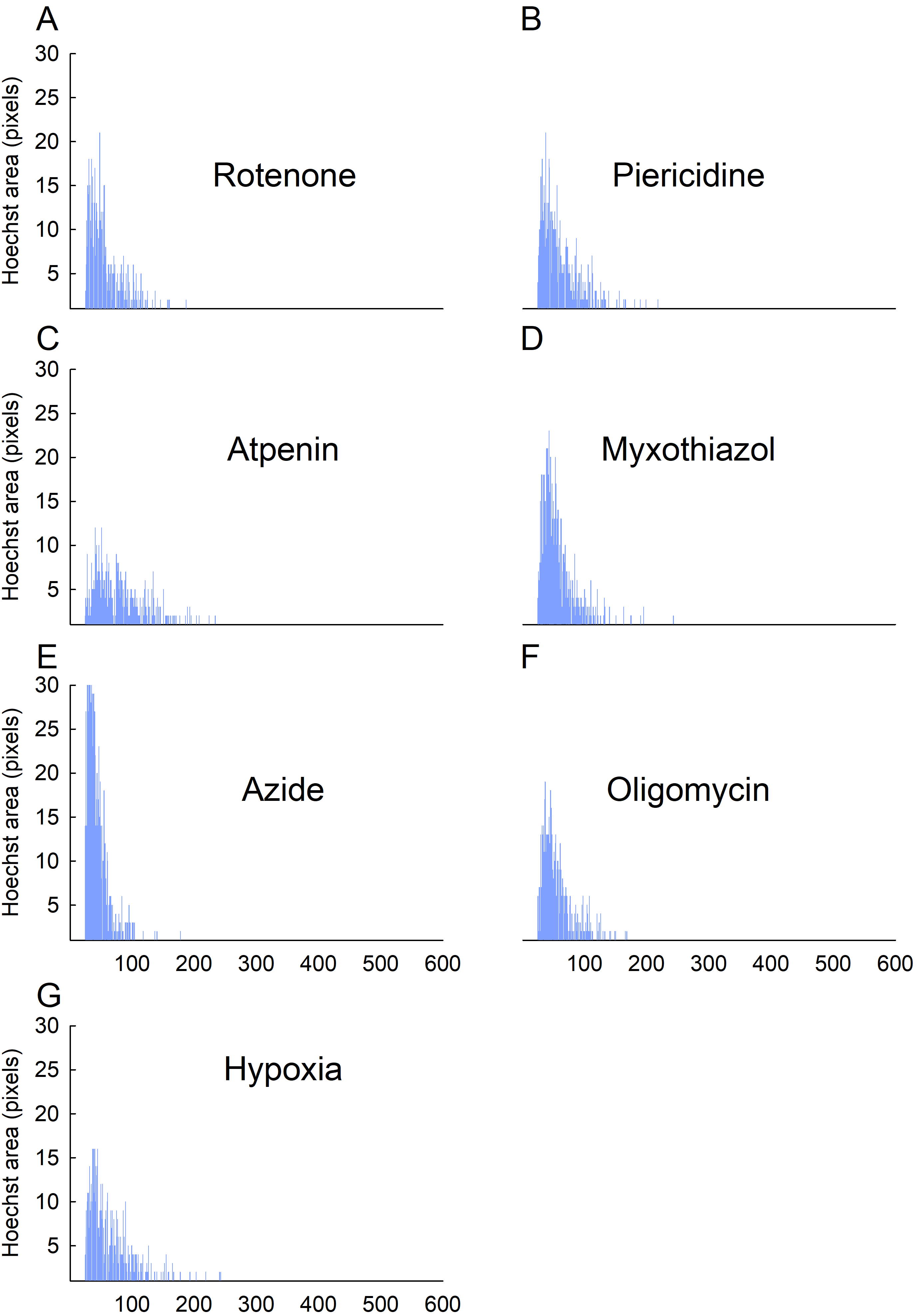

Supplement: Supplementary file 10 — Supplementary Figure 10. [file 41598_2023_37677_MOESM10_ESM.tif]
