# Supplementary material for: Viability of HepG2 and MCF-7 cells is not correlated with mitochondrial bioenergetics
Source: Sci Rep. 2023 Jul 4;13:10822. doi: 10.1038/s41598-023-37677-x (PMC10319846; doi:10.1038/s41598-023-37677-x)
Supplement: Supplementary file 11 — Supplementary Legends. [file 41598_2023_37677_MOESM11_ESM.docx]

**SUPPLEMENTARY MATERIAL**

Viability of HepG2 and MCF-7 Cells is not Correlated with Mitochondrial Bioenergetics

Judit Doczi^1^, Noemi Karnok^1^, David Bui^1^, Victoria Azarov^1^, Gergely Pallag^1^, Sara Nazarian^1^, Bence Czumbel^1^, Thomas N Seyfried^2^, Christos Chinopoulos^1^

^1^Institute of Biochemistry and Molecular Biology, Department of Biochemistry, Semmelweis University, Budapest,1094, Hungary

^2^Biology Department, Boston College, Chestnut Hill, MA 02467, USA

Correspondence: Dr. Christos Chinopoulos, MD, PhD, DSc, e-mail: chinopoulos.christos@med.semmelweis-univ.hu or prof. Thomas N Seyfried, PhD, E-mail: thomas.seyfried@bc.edu

*Supplementary figure legends:*

**Supplementary Figure 1: Membrane polarisation loss with increasing azide concentrations in anoxia.** A representative trace of membrane potential determination (**A**) with isolated mouse mitochondria. Additions and the depletion of oxygen (anox) are indicated on top: 2 mM ADP, varying concentrations of azide, 250 nM SF 6847 (uncoupler) respectively. Blue trace represents the oxygen concentration; red trace represents the rhodamine 123 fluorescence indicative of membrane potential. Simultaneous plot of the % anoxic membrane potential loss (red) and % remaining cytochrome c oxidase catalytic activity (green) (**B**). *Fl*_anox_, *Fl*_azide_ and *Fl*_unc_ are fluorescence values at the end of anoxic, azide inhibited and uncoupled state respectively. Values greater than 100% mean all the polarisation was lost, but there is a gradual decay in fluorescence over time as can be seen on (**A**) after the uncoupler*. k’*_0 mM_, *k’*_5 mM_ and *k’_c_* are the pseudo first order rate constants at 0 mM, 5 mM and varying concentrations of azide respectively, measured in HepG2 cells. Data points indicate median of 3-5 measurements and error bars indicate 1 standard deviation around the mean.

**Supplementary Figure 2: Effect of combined inhibition of CI+ CV or CIII+CV on *in situ* mitochondrial membrane potential (ΔΨm) in HepG2 (A, B) and MCF-7 cells (C,D)**. Y axis reflects ΔΨm expressed as the mitochondrial intensity of TMRM fluorescence decomposed of its plasma membrane content. Addition of each inhibitor is indicated in the graph and at the end of each experiment full mitochondrial depolarization was achieved by the application of mitochondrial depolarization cocktail (MDC) containing: 1 μM valinomycin, 1 μM SF 6847, 2 μM oligomycin. Data indicate mean of three independent experiments and error bars indicate SEM.

**Supplementary Figure 3: Citrate synthase catalytic activity content of MCF-7 cells over time.** Citrate synthase measured after 24h, 48h or 72h at 37 °C under 5% CO_2_ atmosphere in assay media. Inhibitor treated (grey) and their respective control (black) cells were normalized to protein equivalent. Inhibitors were 5 μM rotenone (**A**), 1 μM atpenin A5 (**B**), 1 μM myxothiazol (**C**), 1 mM azide (**D**), 5 μM oligomycin (**E**)**.** Bars indicate the mean of three biological replicates and error bars indicate 1 standard deviation. *a: p <0.05; *b: p <0.001.

**Supplementary Figure 4: Effect of 24h, 48h and 72h of hypoxia on a hypoxia marker (EF5 adduct) formation in HepG2 and MCF-7 cells.** EF adducts (EF add.) stain appears only during hypoxia. Phase contrast (phase) images verify the presence of cells when there is no EF add. staining.

**Supplementary Figure 5: Metabolic profiling and effects of RC inhibitor additions on mitochondrial and glycolytic activities in HepG2 cells normalized to total cell number.** HepG2 cells were cultured in XF96-well cell culture microplates (Seahorse Bioscience) at a density of 2 × 10^4^ cells/per well and then incubated for 24 h (**A,B**), 48h (**C,D)** and 72h (**E,F**) at 37 °C under 5% CO_2_ atmosphere in assay media containing RC inhibitors at the following concentrations: rotenone 5 µM (ROT), piericidin 1 µM (PIER), pyridaben 1 µM (PRDBN), atpenin 1 µM (ATPN), myxothiazol 1 µM (MYXO), azide 1 mM (AZIDE), oligomycin 5 µM (OLGM) and SF 6847 1 µM (SF). OCR (**A,C,E**) and ECAR (**B, D,F**) values were normalized to total cell number/well in the assay. Data are representative of at least three independent experiments, each additions with 12 statistical replicates and error bars indicate SEM. Glc + Gln: glucose + glutamine, A + R: antimycin and rotenone.

**Supplementary Figure 6: Metabolic profiling and effects of RC inhibitor additions on mitochondrial and glycolytic activities in MCF-7 cells normalized to total cell number.** MCF-7 cells were cultured in XF96-well cell culture microplates (Seahorse Bioscience) at a density of 2 × 10^4^ cells/per well and then incubated for 24 h (**A,B**), 48h (**C,D)** and 72h (**E,F**) at 37 °C under 5% CO_2_ atmosphere in assay media containing RC inhibitors at the following concentrations: rotenone 5 µM (ROT), piericidin 1 µM (PIER), pyridaben 1 µM (PRDBN), atpenin 1 µM (ATPN), myxothiazol 1 µM (MYXO), azide 1 mM (AZIDE), oligomycin 5 µM (OLGM) and SF 6847 1 µM (SF). OCR (**A,C,E**) and ECAR (**B, D,F**) values were normalized to total cell number/well in the assay. Data are representative of at least three independent experiments, each additions with 12 statistical replicates and error bars indicate SEM. Glc + Gln: glucose + glutamine, A + R: antimycin and rotenone.

**Supplementary Figure 7: Titration of the uncoupler SF 6847 as a function of *in situ* mitochondrial membrane potential (ΔΨm) in HepG2 (A) and MCF-7 cells (B).** Y axis reflects ΔΨm expressed as the mitochondrial intensity of TMRM fluorescence decomposed of its plasma membrane content. At the end of each experiment full mitochondrial depolarization was achieved by the application of mitochondrial depolarization cocktail (MDC) containing (in μM): 1 valinomycin, 1 SF 6847, 2 oligomycin. Data indicate mean of three independent experiments and error bars indicate SEM from a total of 724 HepG2 and 1561 MCF-7 cells.

**Supplementary Figure 8: Aspartate supplementation partially restores viability of RC inhibited MCF-7 cancer cells.**(**A**) 24h, 48h and 72h site-specific RC inhibition-induced cytotoxicity of aspartate supplemented MCF-7 cells was assessed using nucleus-based quantification of dead (propidium iodide (PI) positive)/ total (Hoechst 33342 (Hoechst) positive) cells. (**B**) Mean number of MCF-7 cells/field of view after 24h, 48h and 72h site-specific RC inhibition. Bars indicate mean of at least three independent experiments, each addition with 12-32 statistical replicates and error bars indicate SEM. *a: p <0.05; *b: p <0.001. Concentrations of RC inhibitors were identical to those used in the metabolic profiling of MCF-7 cells and see it in details in the legend of main figure 6.

**Supplementary Figure 9: Histograms of Hoechst area (pixels) as function of RC inhibitor indicated in the panel, in HepG2 cells.** All panels share the same y-axis. Results shown are for 72 hours treatment.

**Supplementary Figure 10: Histograms of Hoechst area (pixels) as function of RC inhibitor indicated in the panel, in MCF-7 cells.** All panels share the same y-axis. Results shown are for 72 hours treatment.
